# Structures and Absolute Configurations of Diketopiperazine Alkaloids Chrysopiperazines A–C from the Gorgonian-Derived *Penicillium chrysogenum* Fungus

**DOI:** 10.3390/md17050250

**Published:** 2019-04-26

**Authors:** Wei-Feng Xu, Ning Mao, Xiao-Jia Xue, Yue-Xuan Qi, Mei-Yan Wei, Chang-Yun Wang, Chang-Lun Shao

**Affiliations:** 1 Key Laboratory of Marine Drugs, The Ministry of Education of China, School of Medicine and Pharmacy, Ocean University of China, Qingdao 266003, China; Xuweifeng_u@163.com (W.-F.X.); 13012456579@163.com (N.M.); xiaoxuexiaojia@163.com (X.-J.X.); qiyuexuan9475@163.com (Y.-X.Q.); mywei95@126.com (M.-Y.W.); changyun@ouc.edu.cn (C.-Y.W.); 2 Laboratory for Marine Drugs and Bioproducts, Qingdao National Laboratory for Marine Science and Technology, Qingdao 266200, China; 3 College of Food Science and Engineering, Ocean University of China, Qingdao 266003, China

**Keywords:** diketopiperazine alkaloids, oxepine-containing, *Penicillium chrysogenum*, absolute configurations, VCD method

## Abstract

Three new diketopiperazine alkaloids, including two oxepine-containing diketopiperazines, chrysopiperazines A and B (**1** and **2**), and one quinazoline-containing diketopiperazine, chrysopiperazine C (**5**), together with three known analogues (**3**, **4,** and **6**), were isolated from the gorgonian-derived *Penicillium chrysogenum* fungus. The relative and absolute configurations of C-3 and C-15 in **1** and **2,** C-3 and C-14 in **5** were established by NOE modified Marfey’s analysis and electronic circular dichroism (ECD) calculations. Particularly, the absolute configurations of C-19 in **1** and **3**, which was very challenging to be identified due to the flexible conformation in a short aliphatic chain, were successfully determined by the vibrational circular dichroism (VCD) method, supplying with a reliable and optional method to define the absolute configurations. Additionally, this is the first report on oxepine-containing diketopiperazines from the genus *Penicillium*.

## 1. Introduction

**Figure 1:**
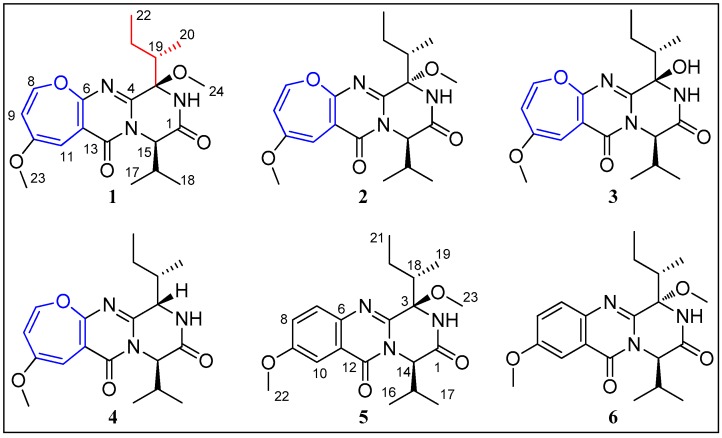
Chemical structures of **1**–**6**.

Diketopiperazines are the smallest cyclic peptides, they are biosynthesised from amino acids by different organisms and are considered to be secondary functional metabolites or side products of terminal peptide cleavage [1]. Oxepine-containing diketopiperazines generally possess a tricyclic core of oxepine−pyrimidinone−diketopiperazine (OPD) derived from different amino acids [2]. Metabolites like that are rarely reported, with only 25 OPD alkaloids having been published so far. Various biological activities such as antifungal [3,4], antibacterial [2], and antiplasmodial [5], have been described. As part of our investigation on structurally novel and biologically active metabolites from marine-derived fungi [6,7,8], two new oxepine-containing diketopiperazines, chrysopiperazines A and B (**1** and **2**); one new quinazoline alkaloid chrysopiperazine C (**5**); and three known analogues, versicoloid B (**3**) [3], versicoloid A (**4**) [3], and versicomide C (**6**) [9] (Figure 1), were isolated from the gorgonian-derived *Penicillium chrysogenum* fungus. The relative and absolute configurations of C-3 and C-15 in **1** and **2**, and C-3 and C-14 in **5** were elucidated by NOE modified Marfey’s analysis and ECD calculations. The absolute configurations of C-19 in **1** and **3**, which were very difficult to be identified, were successfully determined by VCD approach. Herein, we report the isolation and structure elucidation of the new alkaloids **1**, **2,** and **5**.

## 2. Results and Discussion

**Figure 2:**
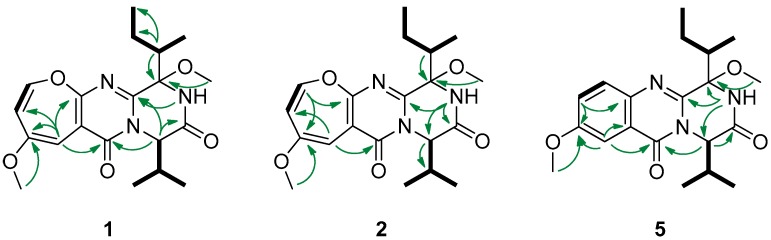
Key ^1^H− ^1^H COSY (bold) and HMBC (arrows) correlations of **1**, **2**, and **5**.

Chrysopiperazine A (**1**) was obtained as yellow gum. Its molecular formula was determined as C_20_H_27_N_3_O_5_ on the basis of the HRESIMS data at *m*/*z* 390.2025 [M + H]^+^ (calcd. for C_20_H_28_N_3_O_5_, 390.2023), indicating 9 degrees of unsaturation. The ^1^H NMR spectrum (Table 1) showed signals of one exchangeable proton [δ_H_ 7.04 (s)]; three unsaturated protons [δ_H_ 6.20 (d, *J* = 6.0 Hz), 5.80 (d, *J* = 1.4 Hz), and 5.53 (dd, *J* = 6.0, 1.4 Hz)]; two methoxyl groups [δ_H_ 3.72 (s) and 3.26 (s)]; and four methyl groups [δ_H_ 1.15 (d, *J* = 7.0 Hz), 1.04 (t, *J* = 7.0 Hz), 1.00 (d, *J* = 6.9 Hz), and 0.92 (d, *J* = 7.2 Hz)]. The ^13^C NMR data of **1** disclosed 20 carbon signals: two amide carbonyls, four sp^2^ quaternary carbons, three sp^2^ methines, one oxygen-bearing sp^3^ quaternary carbon, three sp^3^ methines, one sp^3^ methylene, and six sp^3^ methyls. Three olefinic signals (δ_H/C_ 6.20/144.4, 5.80/94.8, and 5.53/115.8) were typical for an oxepine ring. Detailed analysis of the 1D and 2D NMR spectra of **1** revealed that they were very similar to those of versicoloid A [3]. The key difference was the addition of a methoxyl group (δ_H/C_ 3.26/51.2) at OH-3 in **1**. This deduction was further supported by the COSY and HMBC correlations (Figure 2). Thus, the planar structure of **1** was identified as drawn in Figure 1. 

Chrysopiperazine B (**2**) was isolated as yellow gum, with the same molecular formula, C_20_H_27_N_3_O_5_, as **1** by analysis of the HRESIMS ion peak at *m*/*z* 390.2018 [M + H]^+^ (calcd. for C_20_H_28_N_3_O_5_, 390.2023). The NMR data of **2** (Table 1) were similar to those of **1**, with significant differences in the chemical shift of the methoxyl group (H_3_-24, δ_H_ 2.96 in **2** VS 3.26 in **1**). Detailed analysis of the spectroscopic data (Figure 2) deduced the same planar structure for **2** as for **1**.

Chrysopiperazine C (**5**) was obtained as colorless gum with the molecular formula of C_20_H_27_N_3_O_4_ on the basis of its HRESIMS at 374.2075 [M + H]^+^ (calcd. for C_20_H_28_N_3_O_4_, 374.2074). The NMR data of **5** (Table 1) were similar to those of **1**, with obvious differences in the chemical shift of 1,2,4- trisubstituted aromatic ring [δ_C/H_ C/H-7, C/H-8, C/H-10, 129.4/7.66 (d, *J* = 9.0 Hz), 125.0/7.38 (dd, *J* = 9.0, 2.9 Hz), 106.2/7.65 (d, *J* = 2.9 Hz) in **5** VS C/H-8, C/H-9, C/H-11, 144.4/6.20 (d, *J* = 6.0 Hz), 115.8/5.53 (dd, *J* = 6.0, 1.4 Hz), 94.8/5.80 (d, *J* = 1.4 Hz) in **1**]. Detailed analysis of the spectroscopic data (Figure 2) of **5** established the planar structure as drawn in Figure 1. Chrysopiperazine A (**1**) could be an oxidation product of chrysopiperazine C (**5**) [2].

**Figure 3:**
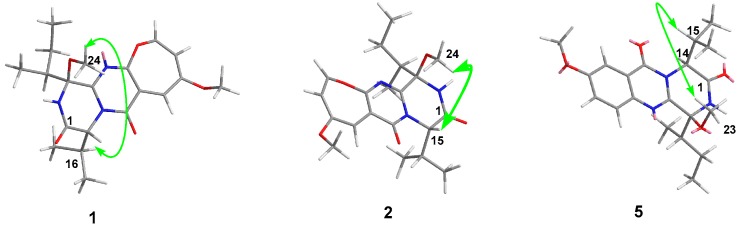
Key NOE correlations of **1**, **2**, and **5**.

The relative configurations of C-3 and C-15 in **1** and **2**, and C-3 and C-14 in **5** were assigned by the analyses of selective 1D NOE data (Figure 3). In the NOE experiment of **1**, the irradiation of H-16 (δ_H_ 2.45) resulted in the enhancement of H_3_-24 (δ_H_ 3.26), which suggested that H-16 and H_3_-24 should be placed on same sides of the ring. In **2**, the irradiation of H-15 (δ_H_ 5.03) resulted in the enhancement of the signal for H_3_-24 (δ_H_ 2.96), which suggested that H-15 and H_3_-24 should be cis-oriented. Similarly, the selective NOE experiments of **5** indicated that H-15 and H_3_-23 were cis-oriented by the irradiation of H-15 (δ_H_ 2.53), which resulted in the enhancement of the signal for H_3_-23 (δ_H_ 3.27). 

To determine the absolute configuration of the valine residue, **1** was hydrolyzed with 6.0 M HCl at 100 °C for 12h and derivatized with Marfey’s reagent [10], followed by comparative HPLC analysis with derivatized standard D and L-amino acids. Surprisingly, an L:D valine ratio of 1:6.7 was observed. Because the NMR spectra did not show a mixture of C-15 epimers, a series of acid hydrolysis conditions were further conducted and hydrolysis results are summarized (Supplementary Materials Figure S24). Only D valine was attained with the modest hydrolysis conditions (6 M HCl at 80 °C for 4h). With increasing hydrolysis time and temperature (6 M HCl at 110 °C for 36h), both L and D-valine residues were observed (L/D-valine = 1:2.4). The above results revealed that the configuration of C-15 was *R*, which originated from the D-valine. The absolute configurations of C-15 in **2** and **3**, and C-14 in **5**, were also determined as *R* by Marfey’s method. Since the relative configurations of C-3 and C-15 in **1**, **2**, and **3**, and C-3 and C-14 in **5** were confirmed, therefore, the absolute configurations of the diketopiperazine ring were determined as 3*R* and 15*R* in **1**, 3*S* and 15*R* in **2**, 3*R* and 15*R* in **3**, and 3*R* and 14*R* in **5**, respectively. 

**Figure 4:**
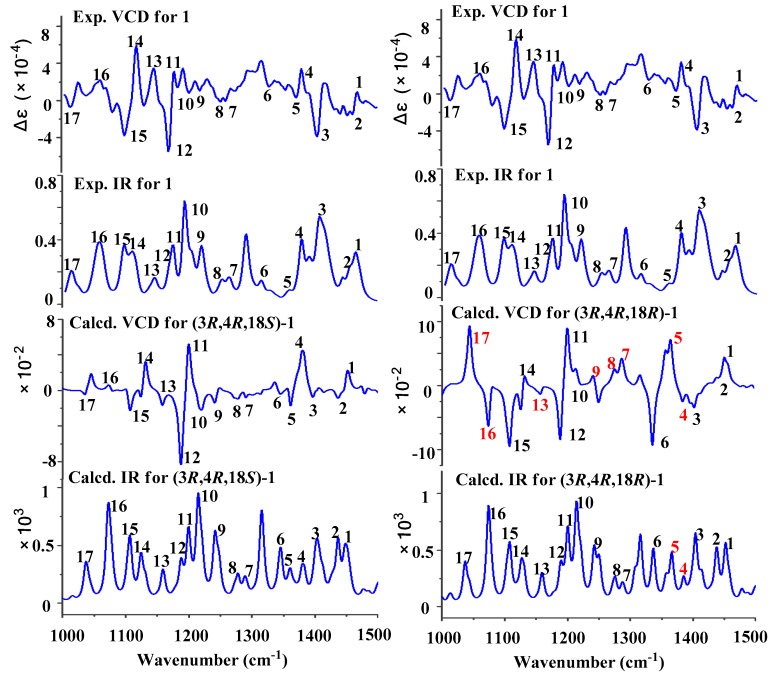
Experimental and calculated VCD/IR spectra of **1**.

The absolute configurations of C-15 in **1** and **3** were further confirmed by comparing experimental results with that of the computed ECD (Supplementary Materials Figure S28). By comparison, the ECD spectrums of **1**, **2** and **3**, with positive (around 220 nm) first and negative (around 250 nm) second Cotton effects, indicated that the configurations at C-15 can greatly influence the ECD Cotton effects; on the opposite side, the configurations at C-3 have little influence on the ECD Cotton effects. It should be mentioned that it is very challenging to identify the configurations of the chiral centers in flexible positions. Until now, the absolute configurations of C-19 in **1**, **2**, and **3**, and C-18 in **5** have not been assigned by the above methods. Based on the above data, two possible absolute configurations, (3*R*,15*R*,19*S*)-**1**/**3** and (3*R*,15*R*,19*R*)-**1**/**3**, were present for **1** and **3**, respectively. Firstly, the predicted ECD spectra of two possible structures [(3*R*,15*R*,19*S*)-**1**/**3** and (3*R*,15*R*,19*R*)-**1**/**3**] of **1** and **3** were calculated using the TD-DFT method at the B3LYP/6-311+G(2d,p)//B3LYP/6-31+G(d) level. The results showed that both of the predicted ECD spectra for (3*R*,15*R*,19*S*)-**1**/**3** and (3*R*,15*R*,19*R*)-**1**/**3** matched well with the measured ECD spectra of **1** and **3** (Supplementary Materials Figure S28), suggesting that it was hard to determine the absolute configurations of C-19 in the side chains of **1** and **3** by ECD method. Recently, the VCD approach has become a robust and reliable alternative for the stereochemical characterizations of natural products [11,12]. Thus, the experimental IR and VCD spectra of **1** and **3** (8.0 and 7.8 mg) were measured in 100 μL of CDCl_3_ using a BioTools dual PEM ChiralIR-2X spectrophotometer. The IR and VCD frequencies of (3*R*,15*R*,19*S*)-**1**/**3** and (3*R*,15*R*,19*R*)-**1**/**3** were calculated at the B3LYP/DGTZVP//B3LYP/DGTZVP level in gas phase and the spectra were used to compare with the experimental IR and VCD spectra of **1** and **3**. As shown in Figure 4 and Supplementary Materials Figure S32, all of the calculated IR and VCD signals of (3*R*,15*R*,19*S*)-**1**/**3** had agreements with the experimental IR and VCD signals of **1**/**3**, indicating the (3*R*,15*R*,19*S*) configuration for **1** and **3**. Based on the common biosynthetic origin, the absolute configurations of C-19 in **2** and **4**, and C-18 in **5** and **6**, were presumed to be the same as that of **1** and **3** for *S*.

Up to now, only 25 OPD alkaloids have been reported from nature sources. They are cinereain (*Botrytis cinerea* [13]), oxepinamides A–G (*Acremonium* sp. [14], *Aspergillus puniceus* [15]), janoxepin *(Aspergillus janus* [5]), brevianamides L, O, and P (*Aspergillus versicolor* [16,17]), protuboxepins A–D, and F–G (*Aspergillus* sp. [18,19], *Aspergillus versicolor* [20]), dihydrocinereain (*Aspergillus carneus* [21]), varioxepine A (*Paecilomyces variotii* [2]), varioloids A and B (*Paecilomyces variotii* [4]), versicoloids A and B (*Aspergillus versicolor* [3]), and versicomide D (*Aspergillus versicolor* [9]). To the best of our knowledge, this is the first report of OPD alkaloids from the genus Penicillium. Because of the configurational flexibility of the isoleucine residue, only 5 of the 13 compounds were assigned for their absolute configurations of C-19 (Table 2). It was reported that the absolute configuration of C-19 could be determined by single-crystal X-ray difraction analysis (oxepinamide E [15] and protuboxepin C [19]) and Marfey’s method (versicomide D [9]), and deduced by the biosynthetic pathway (oxepinamide F [15] and protuboxepin D [19]). However, the oily compounds were difficult to be cultured single crystals for X-ray analysis, and Marfey’s method was also limited to be used due to the oxidation of C-3. In this paper, the efficient VCD method was successfully applied to elucidate the absolute configuration of C-19 for the first time. This provides a reliable and optional method to define the absolute configurations of the analogues.

Compounds **1** and **3** were evaluated for the antibacterial activity against *Escherichia coli*, *Staphylococcus aureus*, *Pseudomonas aeruginosa*, *Photobacterium halotolerans*, and *Enterobacter cloacae*, and the antifungal activity against *Canidia* *albicans*. However, they were inactive at the concentration of 50 μM.

## 3. Materials and Methods 

### 3.1. General Experimental Procedures

Optical rotations were measured on a JASCO P-1020 digital polarimeter ((JASCO Ltd., Tokyo, Japan) at 25 °C with MeOH as solvent. UV spectra were recorded on a Beckman DU 640 spectrophotometer (Beckman Instruments Ltd., Brea, CA, USA). Circular dichroism spectra were recorded with a Jasco J-815-150S circular dichroism spectrometer (JASCO Ltd., Tokyo, Japan). Vibrational circular dichroism spectra were measured on a BioTools ChiralIR-2X spectrophotometer (BioTools, Inc., Jupiter, FL, USA). ^1^H, ^13^C, NOE NMR, and 2D-NMR spectra were recorded at 500 MHz for ^1^H and 125 MHz for ^13^C on a JEOL JEM-ECP NMR spectrometer (JEOL Ltd., Tokyo, Japan). Chemical shifts (*δ*) are reported in ppm, using TMS as the internal standard, and coupling constants (*J*) are in hertz (Hz). High-resolution electrospray ionization mass spectrometry (HRESIMS) spectra were measured on a Micromass Q-TOF mass spectrometer (Waters Ltd., Beverly, MA, USA). High-performance liquid chromatography (HPLC) separation was performed using a Hitachi L-2000 prep-HPLC system coupled with a Hitachi L-2455 photodiode array detector (Hitachi, Tokyo, Japan). The semipreparative HPLC column used was a Kromasil C18 column (Akzo Nobel, Amsterdam, Holland, 7 μm, 10 × 250 mm). Silica gel (100–200 and 200–300 mesh) (Qingdao Haiyang Chemical Co., Ltd., Qingdao, China), Sephadex LH-20 (Amersham Biosciences Inc., Piscataway, NJ, USA), and octadecylsilyl silica gel (45–60 μm) (Merck KGaA, Darmstadt, Germany) were used for column chromatography. Thin layer chromatography (TLC) was precoated with silica gel GF 254 plates (Yantai Zi Fu Chemical Co., Ltd., Yantai, China).

### 3.2. Biological Material

The fungal strain *P. chrysogenum* (CHNSCLM-0019) was isolated from a piece of fresh sample of gorgonian *Dichotella gemmacea* collected in the South China Sea. The fungus was identified as *P. chrysogenum* using its morphological traits and a molecular protocol by DNA amplification and sequencing of the ITS region. The strain was deposited in the Key Laboratory of Marine Drugs, School of Medicine and Pharmacy, Ocean University of China, Qingdao, P.R. China, with the GenBank (NCBI) access number MK696221.

### 3.3. Extraction and Isolation

The fungal strain *P. chrysogenum* (CHNSCLM-0019) was grown on solid media in 100 Erlenmeyer flasks (1 L) each containing 70 g of rice, 1.2 g of glucose, 0.36 g of peptone, 0.84 g of yeast extract, 120 mL of water, and 3.6 g of sea salt. The fungus were fermented at 28 °C for 6 weeks before harvest.

The fermented solid medium was extracted five times with a mixture of 350 mL CH_2_Cl_2_/MeOH (1:1) with each Erlenmeyer flask. The organic extracts were evaporated under vacuum to recovered organic solvents and then partitioned with EtOAc. The combined EtOAc solution was evaporated to dryness under a vacuum to afford the crude extract (80.0 g). The whole extract was subjected to vacuum-liquid chromatography (VLC) on a silica gel (200−300 mesh) column using a gradient of CH_2_Cl_2_−MeOH (100:0 to 0:100, *v*/*v*) to yield four subfractions (Fr. A−Fr. D). Fr. B was subjected to column chromatography on silica gel (200−300 mesh) eluted with petroleum ether/EtOAc (100: 0 to 0: 100, *v*/*v*) to afford six subfractions (Fr. B1−Fr. B6). Fr. B2 was subjected to CC over reverse-phase silica gel (ODS) using step-gradient elution with MeOH−H_2_O (25:75 to 100:0, *v*/*v*) to separate into nine subfractions (Fr. B2-1−Fr. B2-13). Fr. B2-5 was applied to Sephadex LH-20 (MeOH) and further purified by HPLC using a C18 (Kromasil 7 μm, 10 × 25 mm) column at a flow rate of 2.0 mL/min (60% MeOH/H_2_O) to yield **4** (1.0 mg, *t*_R_ = 23.5 min), **2** (4.0 mg, *t*_R_ = 31.2 min), and **1** (8.5 mg, *t*_R_ = 34.0min). Fr. B2-5 was purified by semi-preparative HPLC using MeOH−H_2_O (65%) as eluent to yield **6** (4.2 mg, *t*_R_ = 23.2 min). Fr. B2-6 was separated by semi-preparative HPLC (MeOH: H_2_O = 75:25, *v*/*v*) to give compound **5** (4.4 mg, *t*_R_ = 19.3 min). Fr. B3 was applied to Sephadex LH-20 chromatography eluting with mixtures of CH_2_Cl_2_/MeOH (1:1) to obtain five subfractions (Fr. B3-1−Fr. B3-5). Fr. B3-2 was chromatographed on a ODS column using MeOH−H_2_O (50%) and then subjected to semi-preparative HPLC (60% MeOH/H_2_O) to yield **3** (8.0 mg, *t*_R_ = 32.8 min).

#### 3.3.1. Chrysopiperazine A (**1**)

Yellow gum; $[\alpha {]}_{D}^{20}$: −84.8 (*c* 0.45, MeOH); UV (MeOH) λ_max_ (log *ε*): 254 (3.72), 355 (3.58) nm; CD (MeOH) *λ* max (Δ*ε*): 220 (+78.5), 253 (−76.4); ^1^H NMR and ^13^C NMR data, see Table 1; HRESIMS *m*/*z* 358.1764 [M − OCH_3_]^+^ (calcd. for C_19_H_24_N_3_O_4_, 358.1761), 390.2025 [M + H]^+^ (calcd. for C_20_H_28_N_3_O_5_, 390.2023).

#### 3.3.2. Chrysopiperazine B (**2**)

Yellow gum; $[\alpha {]}_{D}^{20}$: −10.7 (*c* 0.38, MeOH); UV (MeOH) λ_max_ (log *ε*): 257 (3.74), 357 (3.62) nm; CD (MeOH) *λ* max (Δ*ε*): 220 (+78.5), 252 (−56.8); ^1^H NMR and ^13^C NMR data, see Table 1; HRESIMS *m*/*z* 358.1758 [M − OCH_3_]^+^ (calcd. for C_19_H_24_N_3_O_4_, 358.1761), 390.2018 [M + H] ^+^ (calcd. for C_20_H_28_N_3_O_5_, 390.2023).

#### 3.3.3. Chrysopiperazine C (**5**)

Colorless gum; $[\alpha {]}_{D}^{20}$: −24.3 (*c* 0.2, MeOH); UV (MeOH) *λ*_max_ (log *ε*): 225 (4.22), 282 (3.68), 324 (3.35) nm; CD (MeOH) *λ* max (Δ*ε*): 220 (+27.3), 237 (−78.5), 282 (+7.6), 326 (−9.3);^1^H NMR and ^13^C NMR data, see Table 1; HRESIMS *m*/*z* 342.1811 [M − OCH_3_]^+^ (calcd. for C_19_H_24_N_3_O_3_, 342.1812), 374.2075 [M + H]^+^ (calcd. for C_20_H_28_N_3_O_4_, 374.2074).

### 3.4. Preparation and Analysis of Marfey’s Derivatives

Compounds (0.2 mg) were separately hydrolyzed for 30 min to 36 h in 6.0 M HCl under different temperatures (ranging from 40 °C to 110 °C). After cooling, the solutions were evaporated to dryness and redissolved in H_2_O (50 μL). The above hydrolysis solutions were added 200 μL of 0.5% (*w*/*v*) FDAA (Marfey’s reagent; 1-fluoro-2,4-dinitrophenyl-5-L-alanine amide) in acetone solution. After the addition of 20 μL 1 M NaHCO_3_ solution, the mixture was incubated at 45 °C for 40 min. The reactions were stopped by the addition of 20 μL of 2 M HCl. The solvents were evaporated to dryness, and the resulting residues were dissolved in 20 μL of MeOH. Separately, L-Val and D-Val were derivatized with FDAA in the same manner as that of natural products. The standard amino acid derivatives were analyzed by HPLC (Kromasil 10 × 250 mm, 7 μm, 1.0 mL/min) with linear gradient: (A) CH_3_CN and (B) H_2_O with 0.1% trifluoroacetic acid 30–50% A (0–30 min), 50–100% A (30–35 min), 100% A (35–37 min), 100–30% A (37–40 min), detected at 340 nm. The retention times of the FDAA derivatives of L-Val and D-Val were 14.4 and 18.2 min, respectively.

### 3.5. Bioactivity Assay

The antibacterial activity activity of **1** and **3** against *Escherichia coli*, *Staphylococcus aureus*, *Pseudomonas aeruginosa*, *Photobacterium halotolerans*, and *Enterobacter cloacae*, and antifungal activity against *Canidia albicans* were evaluated by using 96-well microtiter plates [22], with ciprofloxacin and SeaNine 211 as positive controls.

## 4. Conclusions

Three new diketopiperazine alkaloids, chrysopiperazines A−C (**1**–**3**), were isolated from the gorgonian-derived *Penicillium chrysogenum* fungus and their relative and absolute configurations were comprehensively established by NOE modified Marfey’s analysis, ECD and VCD calculations. This research also provides a series of combined methods to define the absolute configurations of complicated natural products.

## Figures and Tables

**Table 1:** ^1^H NMR and ^13^C NMR data of 1–3 ^a^.

Position	1		2		5	
	*δ*_C_	*δ*_H_ (*J* in Hz)	*δ*_C_	*δ*_H_ (*J* in Hz)	*δ*_C_	*δ*_H_ (*J* in Hz)
1	169.2		166.8		169.9	
2		7.04, s		6.10, s		6.61, s
3	88.1		90.4		88.5	
4	149.8		151.2		144.6	
6	158.3		159.5		140.4	
7					129.4	7.66, d (9.0)
8	144.4	6.20, d (6.0)	144.8	6.22, d (6.0)	125.0	7.38, dd (9.0, 2.9)
9	115.8	5.53, dd (6.0, 1.4)	115.4	5.52, dd (6.0, 1.4)	159.1	
10	157.3		157.4		106.2	7.65, d (2.9)
11	94.8	5.80, d (1.4)	94.4	5.78, d (1.4)	121.3	
12	110.5		110.6		161.3	
13	161.8		161.3			
					60.7	5.24, d (8.7)
15	61.0	5.07, d (7.8)	61.2	5.03, d (4.1)	33.4	2.53, m
16	33.3	2.45, m	33.1	2.38, m	19.6	0.95, d (6.8)
17	19.5	1.15, d (7.0)	20.3	1.23, d (7.0)	20.0	1.22, d (6.7)
18	19.7	1.00, d (6.9)	17.8	0.97, d (6.9)	36.2	2.96, m
19	36.4	2.73, m	42.1	2.50, m	10.9	1.07, d (7.0)
20	12.1	0.92, d (7.2)	14.9	0.95, d (6.9)	25.1	1.26, m, 1.00, m
21	24.8	1.11, m	21.4	1.03, m; 1.96, m	12.4	0.93, t (7.0)
22	10.6	1.04, t (7.0)	11.9	1.00, t (6.9)	55.8	3.96, s
23	55.2	3.72, s	55.2	3.73, s	50.9	3.27, s
24	51.2	3.26, s	50.5	2.96, s		

^a^ Measured in CDCl_3_, 500 MHz for ^1^H NMR and 125 MHz for ^13^C NMR.

**Table 2:** OPD alkaloids containing C-19 chiral carbon.

Compd.	Source	Absolute Configuration of C-19	Method
Oxepinamide A^1^^4^	*Acremonium* sp.	not determined	-
Oxepinamide B^1^^4^	*Acremonium* sp.	not determined	-
Brevianamide L^16^	*Aspergillus versicolor*	not determined	-
Brevianamide O^17^	*A. versicolor*	not determined	-
Brevianamide P^17^	*A. versicolor*	not determined	-
Protuboxepin A^18^	*Aspergillus* sp.	not determined	-
Oxepinamide E^15^	*A. puniceus*	*S*	X-ray
Oxepinamide F^15^	*A. puniceus*	*S*	biosynthetic origins
Versicoloid A^3^	*A. versicolor*	not determined	-
Versicoloid B^3^	*A. versicolor*	not determined	-
Versicomide D^9^	*A. versicolor*	*S*	acid hydrolyzed
Protuboxepin C^19^	*Aspergillus* sp.	*S*	X-ray
Protuboxepin D^19^	*Aspergillus* sp.	*S*	biosynthetic origins
Chrysopiperazine A	*P. chrysogenum*	*S*	VCD
Chrysopiperazine B	*P. chrysogenum*	*S*	VCD

## Supplementary Materials

The following are available online at https://www.mdpi.com/1660-3397/17/5/250/s1, Figure S1–S29: 1D (^1^H NMR, ^13^C NMR, and NOE) spectra, and 2D (COSY, HSQC, and HMBC) spectra and HRESIMS of new compounds **1**, **2**, and **5**, CD spectra of **2** and **5**, Marfey’s analysis of **1**–**3**, and **5**, and experimental and calculated ECD and VCD/IR of **1** and **3**, Scheme S1: Reaction of the derivatization with the Marfey’s reagent, Table S1–S8: The coordinate for the lowest-energy conformer in VCD calculations.
