# Supplementary material for: Structures and Absolute Configurations of Diketopiperazine Alkaloids Chrysopiperazines A–C from the Gorgonian-Derived Penicillium chrysogenum Fungus
Source: Mar Drugs. 2019 Apr 26;17(5):250. doi: 10.3390/md17050250 (PMC6562614; doi:10.3390/md17050250)
Supplement: Supplementary file 1 [file marinedrugs-17-00250-s001.pdf]

SUPPLEMENTARY MATERIAL

Structures and Absolute Configurations of Diketopiperazine Alkaloids Chrysopiperazines A−C from the Gorgonian-Derived *Penicillium chrysogenum* Fungus

Wei-Feng Xu [1, 2, †], Ning Mao [1, 2, †], Xiao-Jia Xue [1, 2], Yue-Xuan Qi [1, 2], Mei-Yan Wei [1, 2, 3], Chang-Yun Wang [1, 2] and Chang-Lun Shao [1, 2]*

1 Key Laboratory of Marine Drugs, The Ministry of Education of China, School of Medicine and Pharmacy, Ocean University of China, Qingdao 266003, People's Republic of China.
2 Laboratory for Marine Drugs and Bioproducts, Qingdao National Laboratory for Marine Science and Technology, Qingdao 266200, People's Republic of China
3 College of Food Science and Engineering, Ocean University of China, Qingdao 266003, People's Republic of China
* Correspondence: shaochanglun@163.com (C.-L. Shao)
† These authors contributed equally to this work.

List of supporting information

Figure S1. ^{1}H NMR (500 MHz, $CDCl_3$) spectrum of **1**

Figure S2. ^{13}C NMR (125 MHz, $CDCl_3$) spectrum of **1**

Figure S3. HMQC ($CDCl_3$) spectrum of **1**

Figure S4. ^{1}H–^{1}H COSY ($CDCl_3$) spectrum of **1**

Figure S5. HMBC ($CDCl_3$) spectrum of **1**

Figure S6. 1D NOE ($CDCl_3$) spectrum of **1**

Figure S7. HRESIMS spectrum of **1**

Figure S8. ^{1}H NMR (500 MHz, $CDCl_3$) spectrum of **2**

Figure S9. ^{13}C NMR (125 MHz, $CDCl_3$) spectrum of **2**

Figure S10. HMQC ($CDCl_3$) spectrum of **2**

Figure S11. ^{1}H–^{1}H COSY ($CDCl_3$) spectrum of **2**

Figure S12. HMBC ($CDCl_3$) spectrum of **2**

Figure S13. 1D NOE ($CDCl_3$) spectrum of **2**

Figure S14. HRESIMS spectrum of **2**

Figure S15. CD spectrum of **2**

Figure S16. ^{1}H NMR (500 MHz, $CDCl_3$) spectrum of **5**

Figure S17. ^{13}C NMR (125 MHz, $CDCl_3$) spectrum of **5**

Figure S18. HMQC ($CDCl_3$) spectrum of **5**

Figure S19. ^{1}H–^{1}H COSY ($CDCl_3$) spectrum of **5**

Figure S20. HMBC ($CDCl_3$) spectrum of **5**

Figure S21. 1D NOE ($CDCl_3$) spectrum of **5**

Figure S22. HRESIMS spectrum of **5**

Figure S23. CD spectrum of **5**

Scheme S1. Reaction of the derivatization with the Marfey's reagent

Figure S24. Marfey's analysis of the valine acid in **1** on HPLC

Figure S25. Marfey's analysis of the valine acid in **2** on HPLC

Figure S26. Marfey's analysis of the valine acid in **3** on HPLC

Figure S27. Marfey's analysis of the valine acid in **5** on HPLC

Formatted: Font color: Red

Figure S28. Experimental and calculated ECD spectra of **1** and **3**

Figure S29. Experimental and calculated VCD/IR spectra of **3**

Table S1. The coordinate for the lowest-energy conformer [(3*R*,15*R*,19*S*)**-1**] in ECD calculations

Table S2. The coordinate for the lowest-energy conformer [(3*R*,15*R*,19*R*)**-1**] in ECD calculations

Table S3. The coordinate for the lowest-energy conformer [(3*R*,15*R*,19*S*)**-3**] in ECD calculations

Table S4. The coordinate for the lowest-energy conformer [(3*R*,15*R*,19*R*)**-3**] in ECD calculations

Table S5. The coordinate for the lowest-energy conformer [(3*R*,15*R*,19*S*)**-1**] in VCD calculations

Table S6. The coordinate for the lowest-energy conformer [(3*R*,15*R*,19*R*)**-1**] in VCD calculations

Table S7. The coordinate for the lowest-energy conformer [(3*R*,15*R*,19*S*)**-3**] in VCD calculations

Table S8. The coordinate for the lowest-energy conformer [(3*R*,15*R*,19*R*)**-3**] in VCD calculations

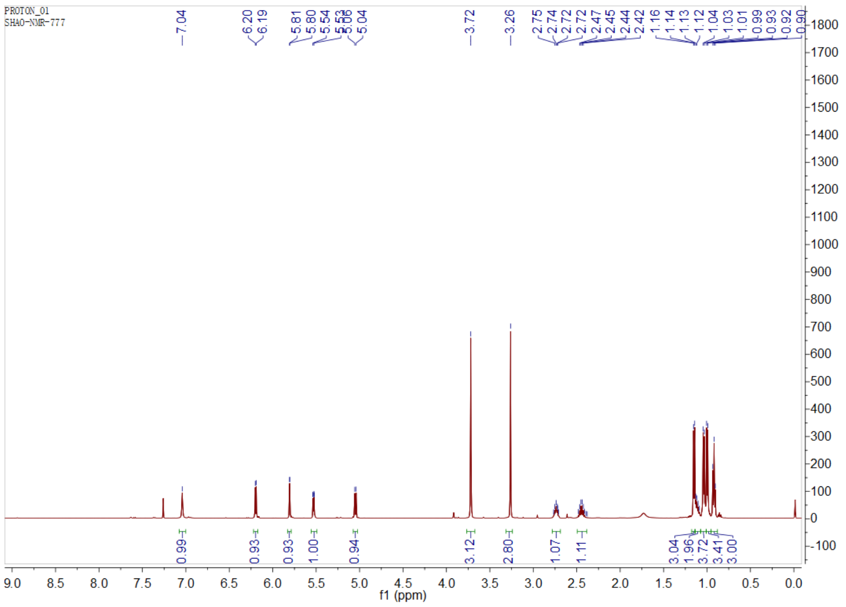

Figure S1. ^{1}H NMR (500 MHz, $CDCl_3$) spectrum of **1**

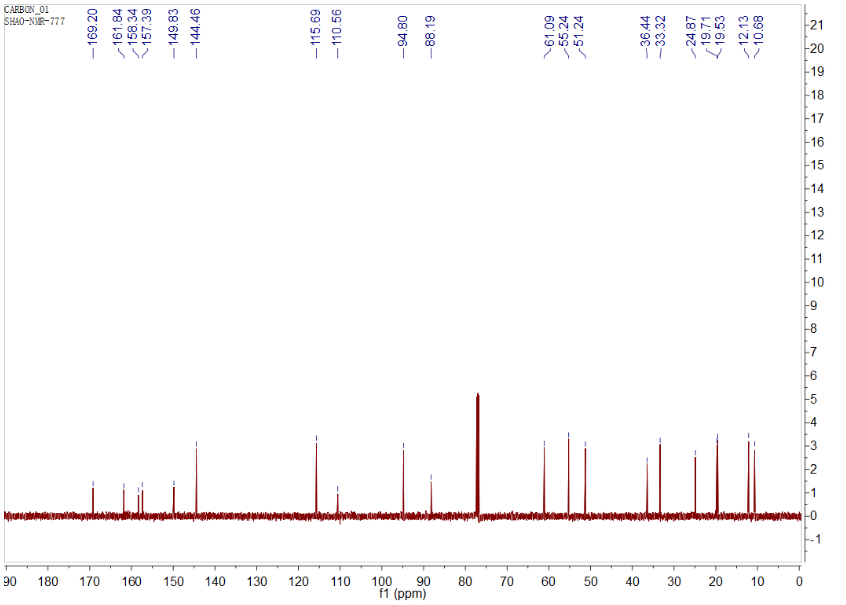

Figure S2. ^{13}C NMR (125 MHz, $CDCl_3$) spectrum of **1**

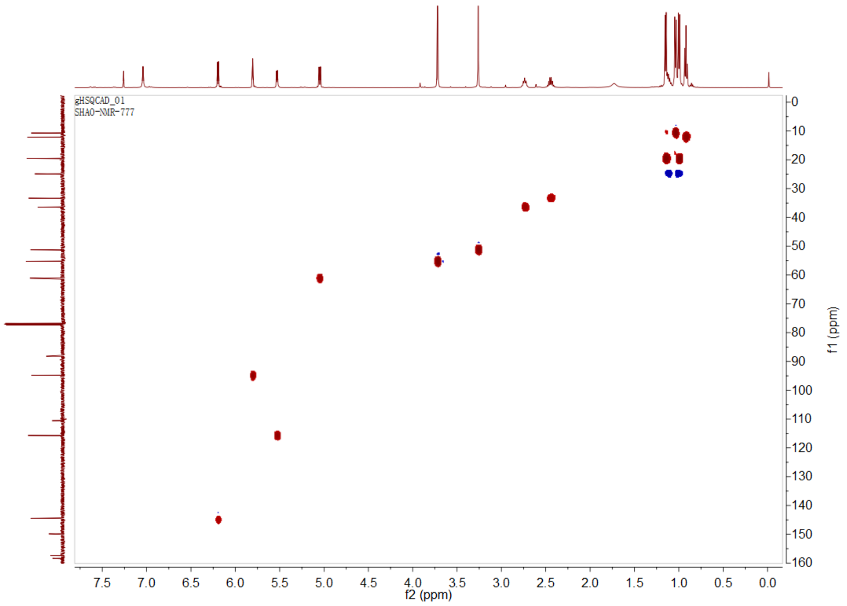

Figure S3. HMQC ($CDCl_3$) spectrum of **1**

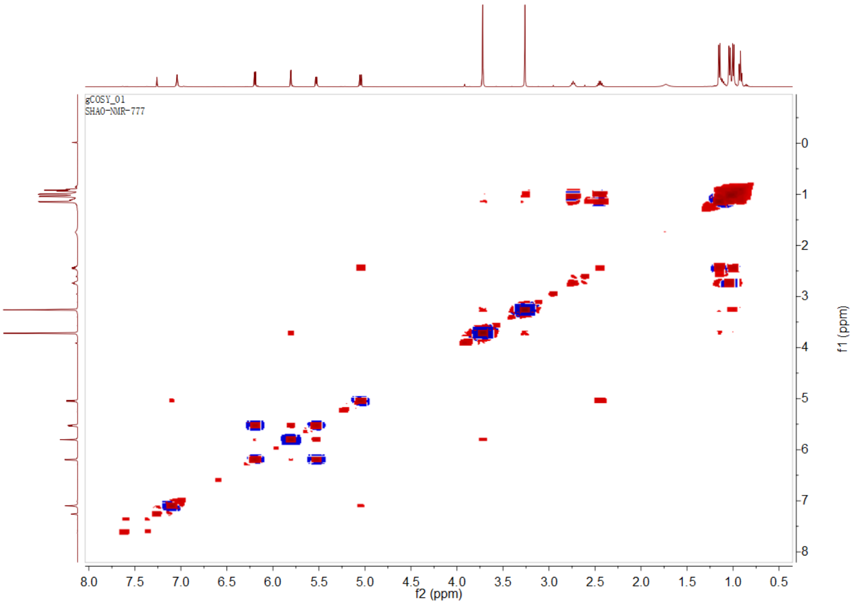

Figure S4. ^{1}H–^{1}H COSY ($CDCl_3$) spectrum of **1**

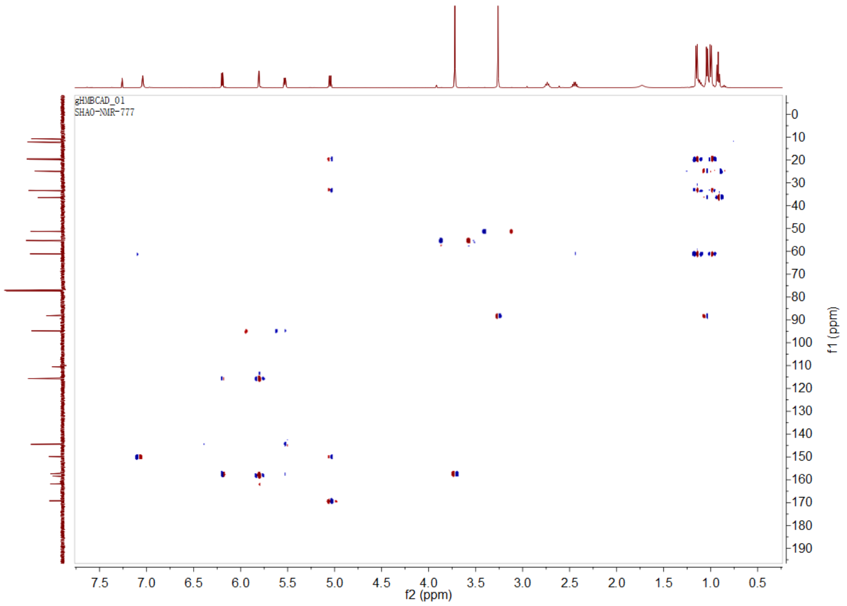

Figure S5. HMBC ($CDCl_3$) spectrum of **1**

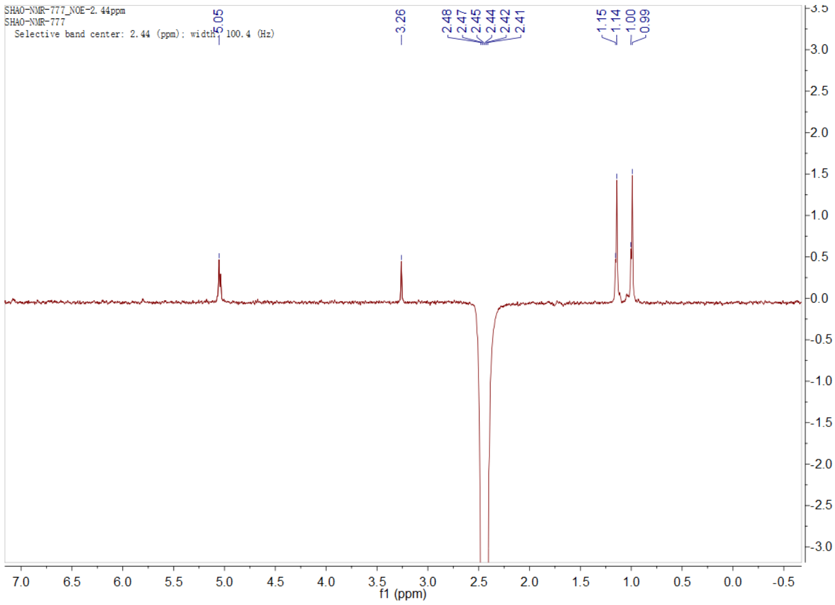

Figure S6. NOE ($CDCl_3$) spectrum of **1**

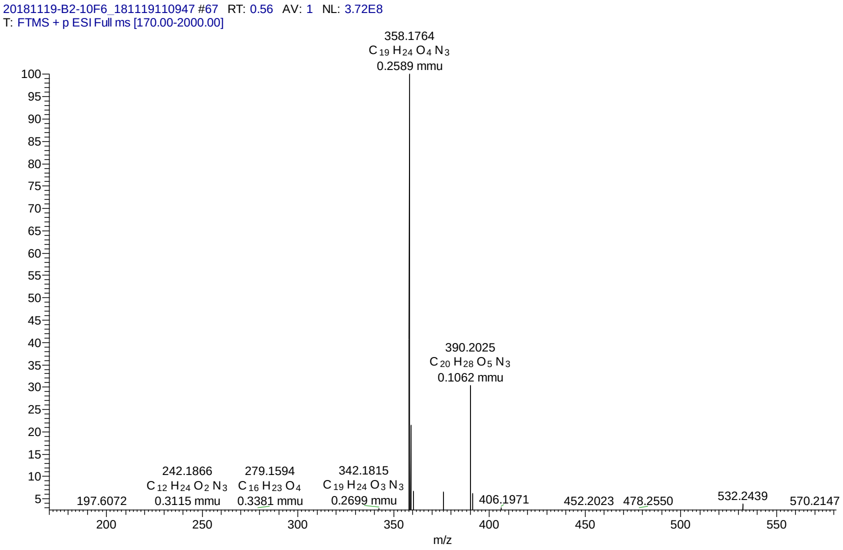

Figure S7. HRESIMS spectrum of **1**

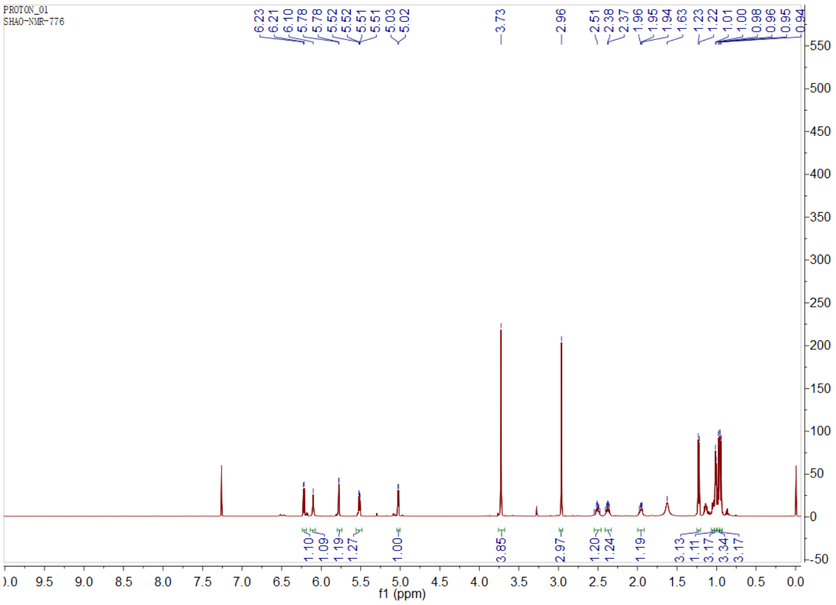

Figure S8. ^1H NMR (500 MHz, $CDCl_3$) spectrum of **2**

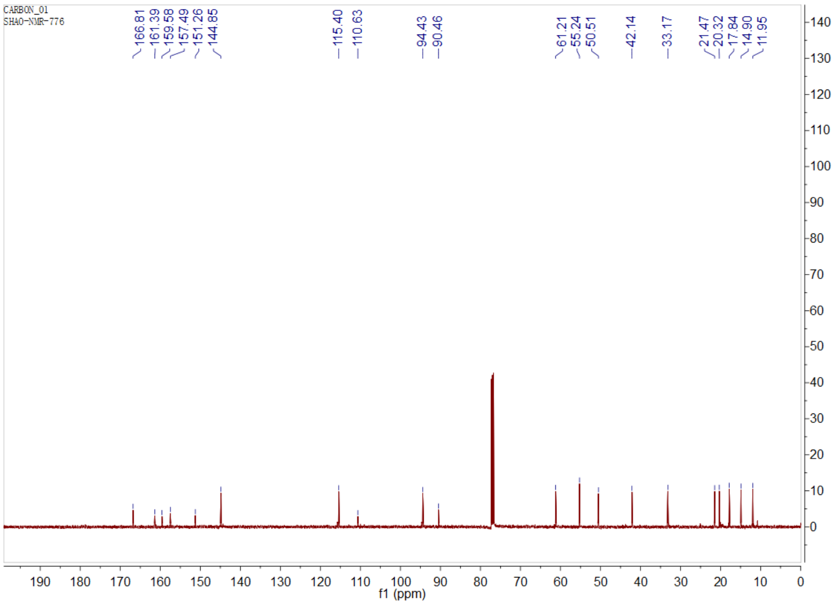

Figure S9. ^{13}C NMR (125 MHz, $CDCl_3$) spectrum of **2**

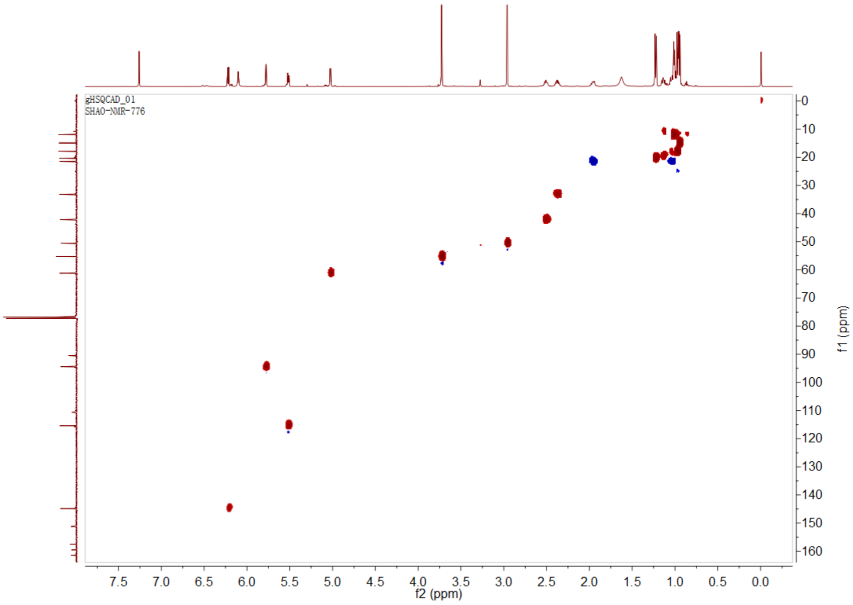

Figure S10. HMQC ($CDCl_3$) spectrum of **2**

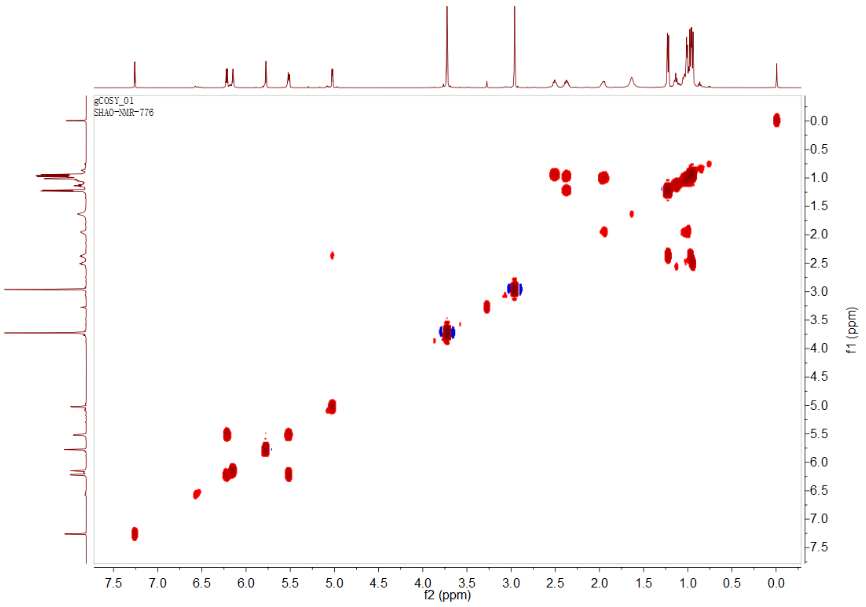

Figure S11. ^1H–^1H COSY ($CDCl_3$) spectrum of **2**

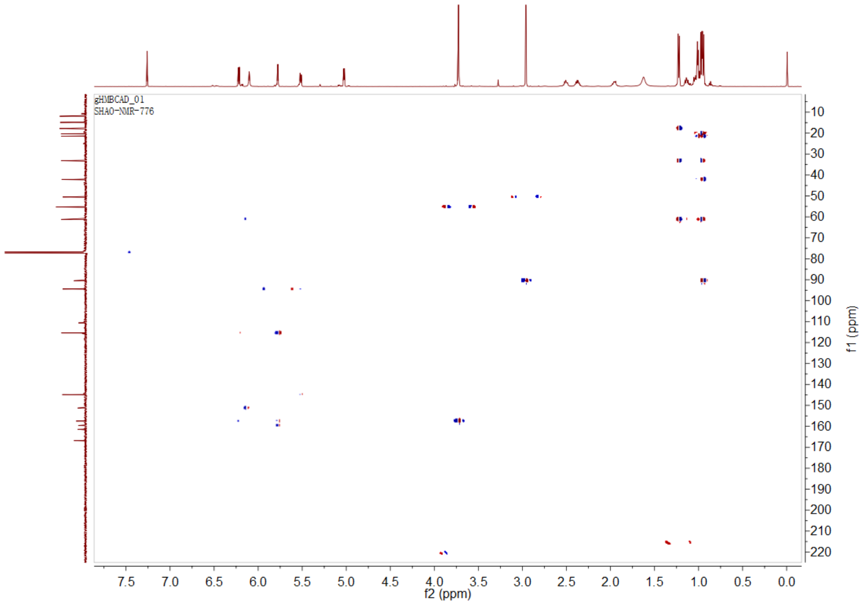

Figure S12. HMBC ($CDCl_3$) spectrum of **2**

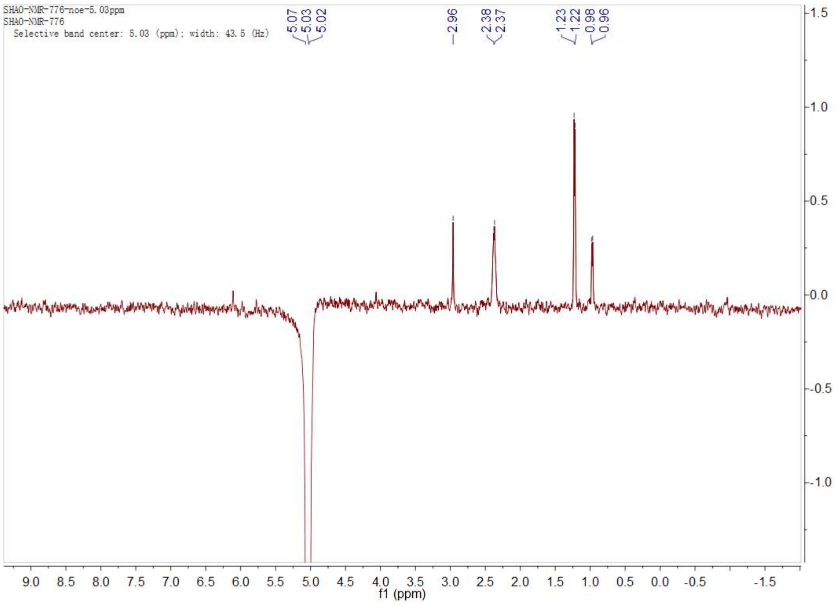

Figure S13. NOE ($CDCl_3$) spectrum of **2**

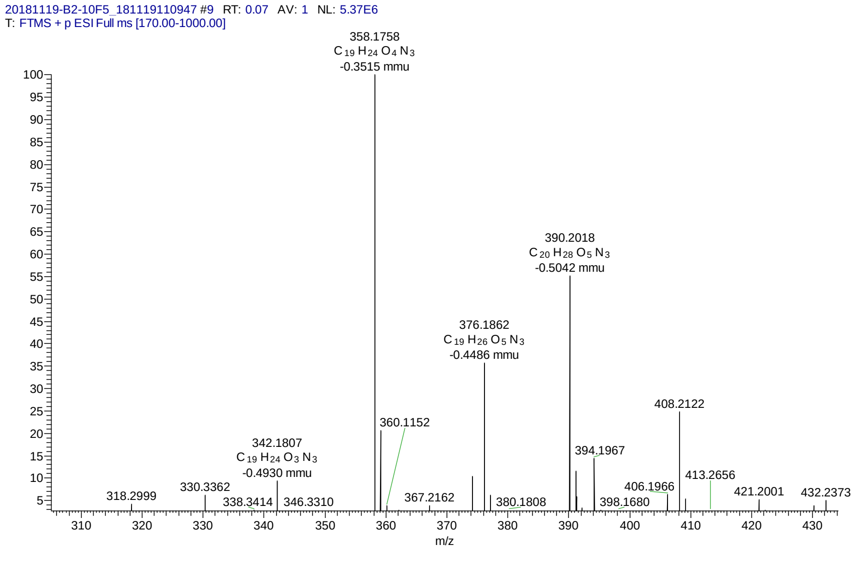

Figure S14. HRESIMS spectrum of **2**

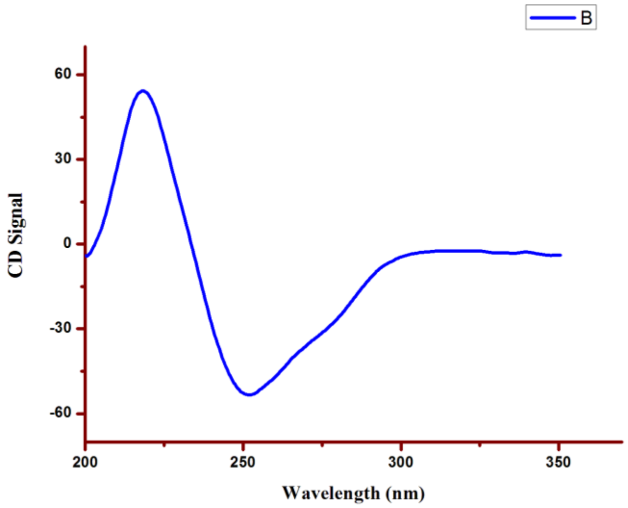

Figure S15. CD spectrum of **2**

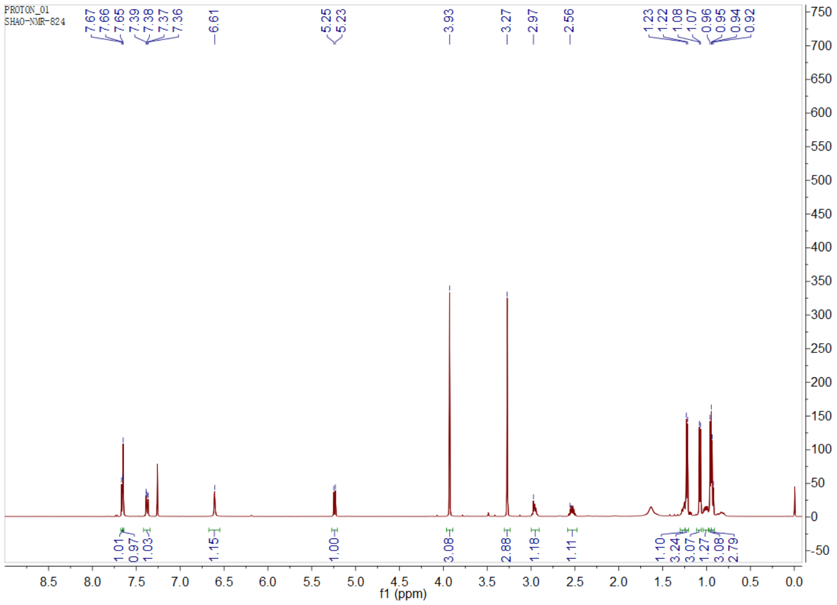

Figure S16. ^{1}H NMR (500 MHz, $CDCl_3$) spectrum of **5**

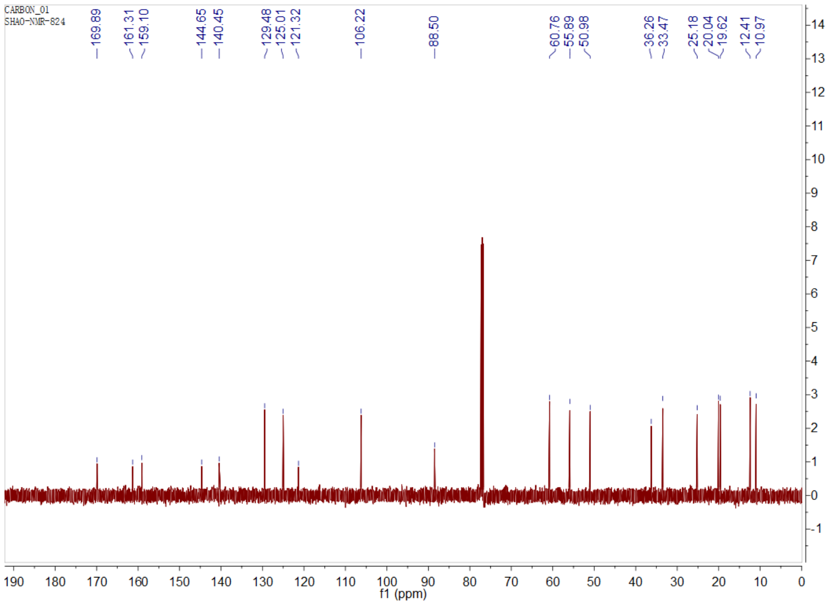

Figure S17. ^{13}C NMR (125 MHz, $CDCl_3$) spectrum of **5**

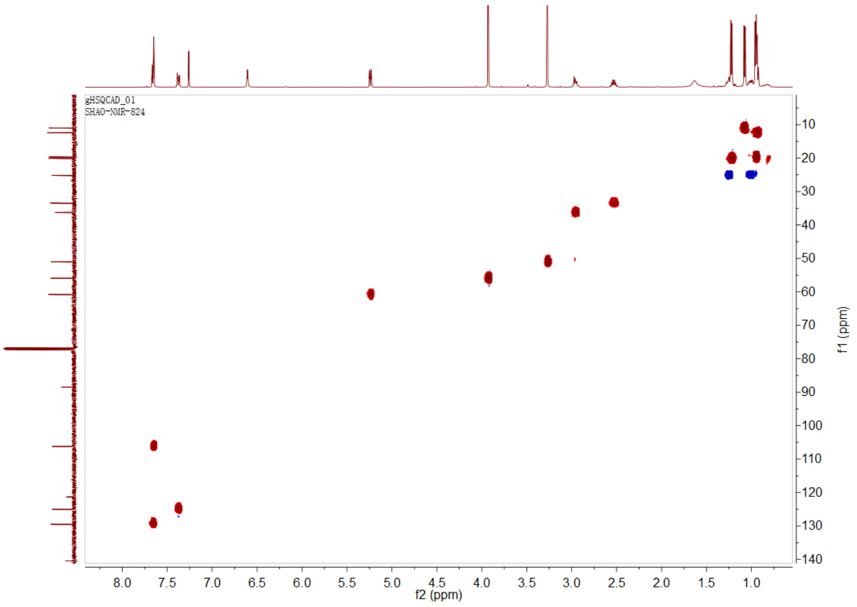

Figure S18. HMQC ($CDCl_3$) spectrum of **5**

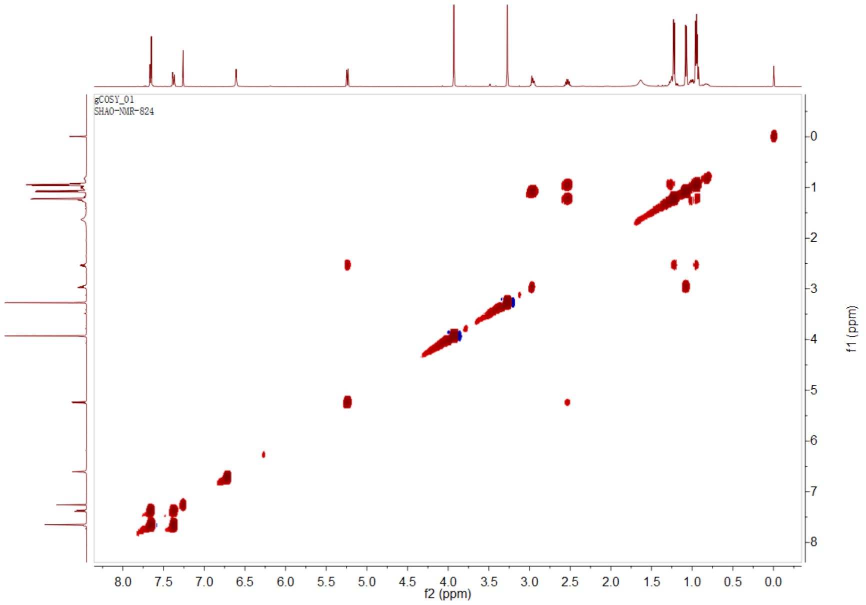

Figure S19. ^1H–^1H COSY ($CDCl_3$) spectrum of **5**

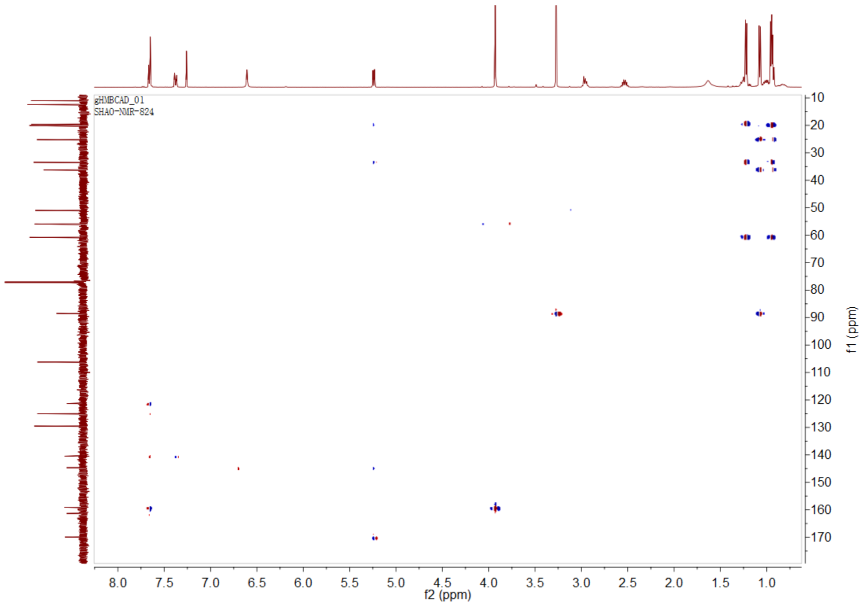

Figure S20. HMBC ($CDCl_3$) spectrum of **5**

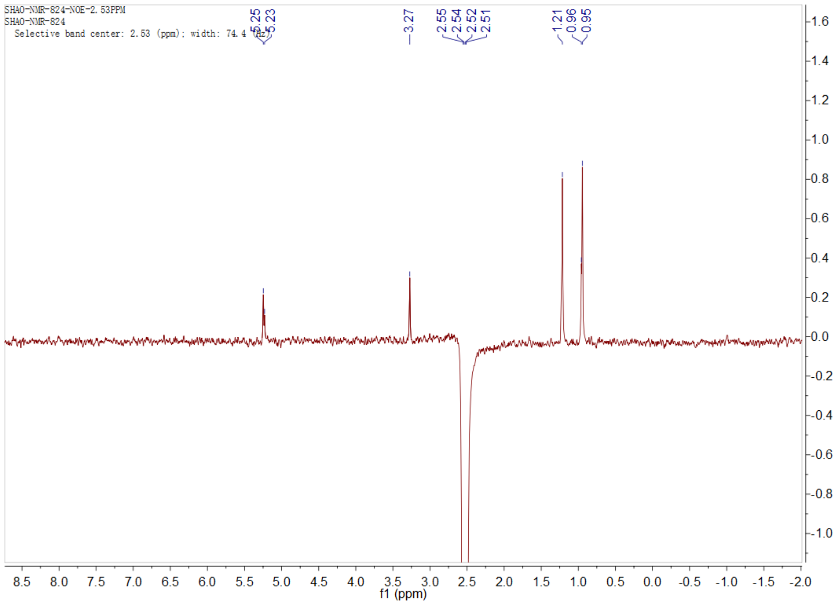

Figure S21. NOE ($CDCl_3$) spectrum of **5**

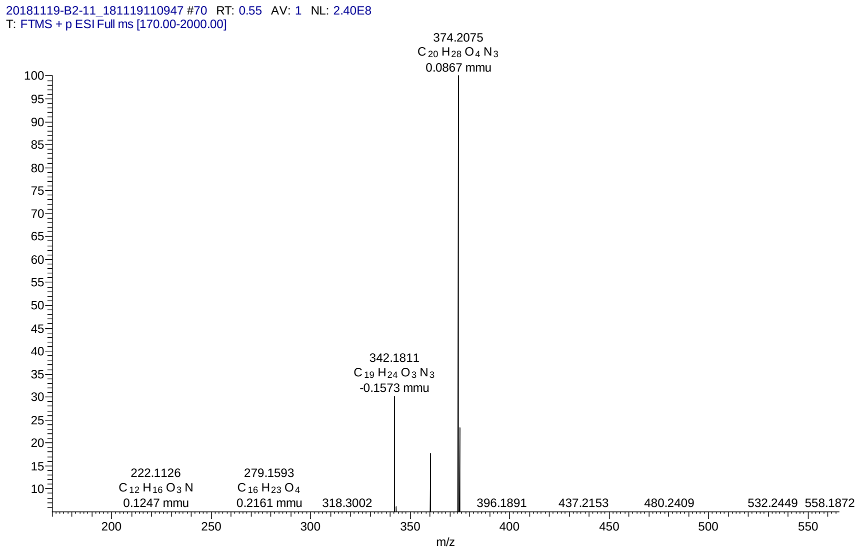

Figure S22. HRESIMS spectrum of **5**

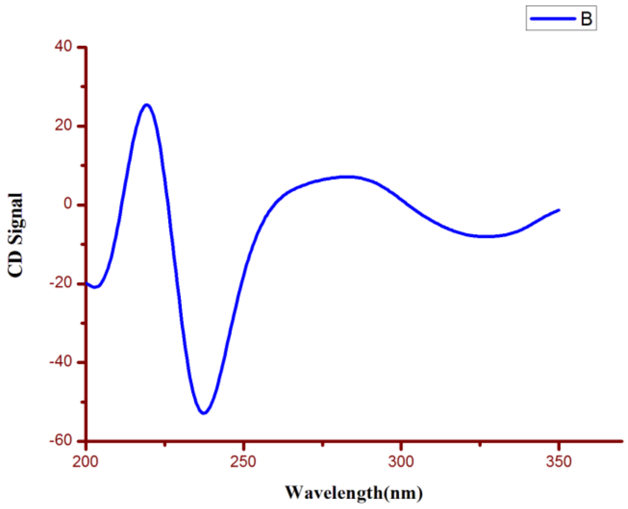

Figure S23. CD spectrum of **5**

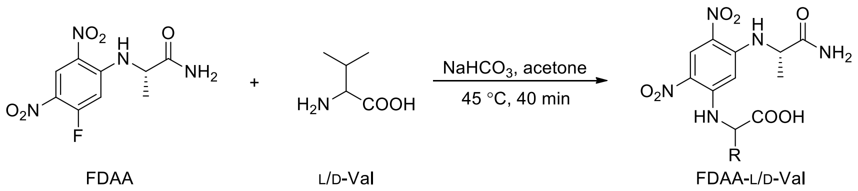

Scheme S1. Reaction of the derivatization with the Marfey's reagent

Field Code Changed

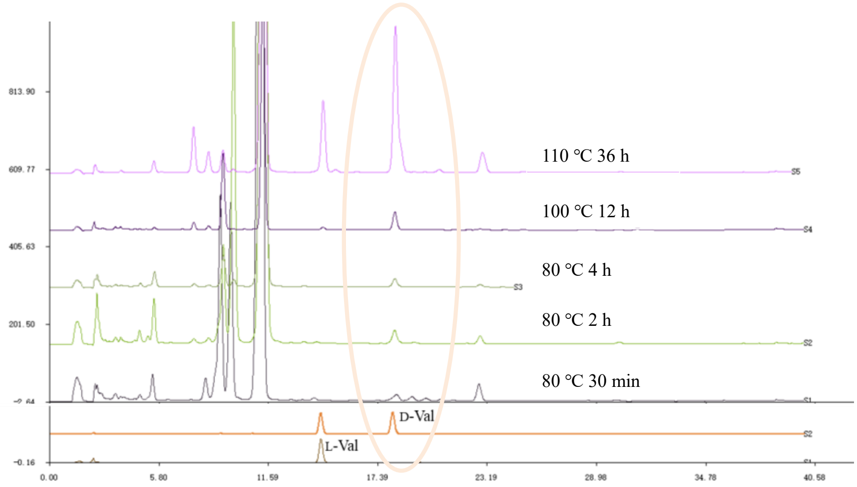

Figure S24. Marfey’s analysis of the valine acid in **1** on HPLC

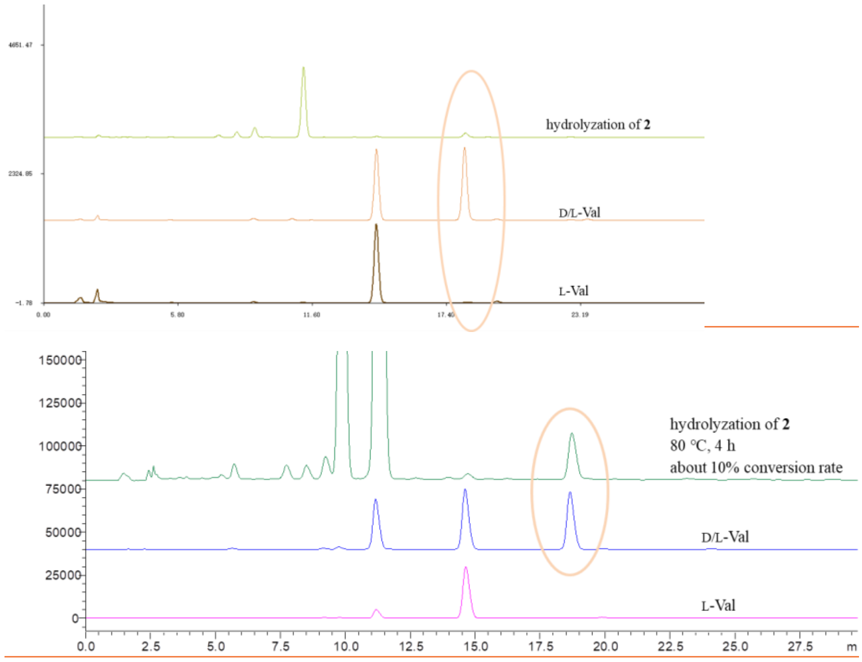

Figure S25. Marfey’s analysis of the valine acid in **2** on HPLC

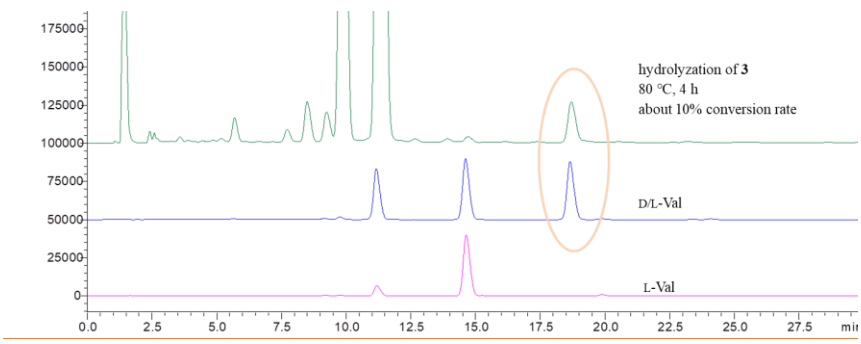

Figure S26. Marfey's analysis of the valine acid in **3** on HPLC

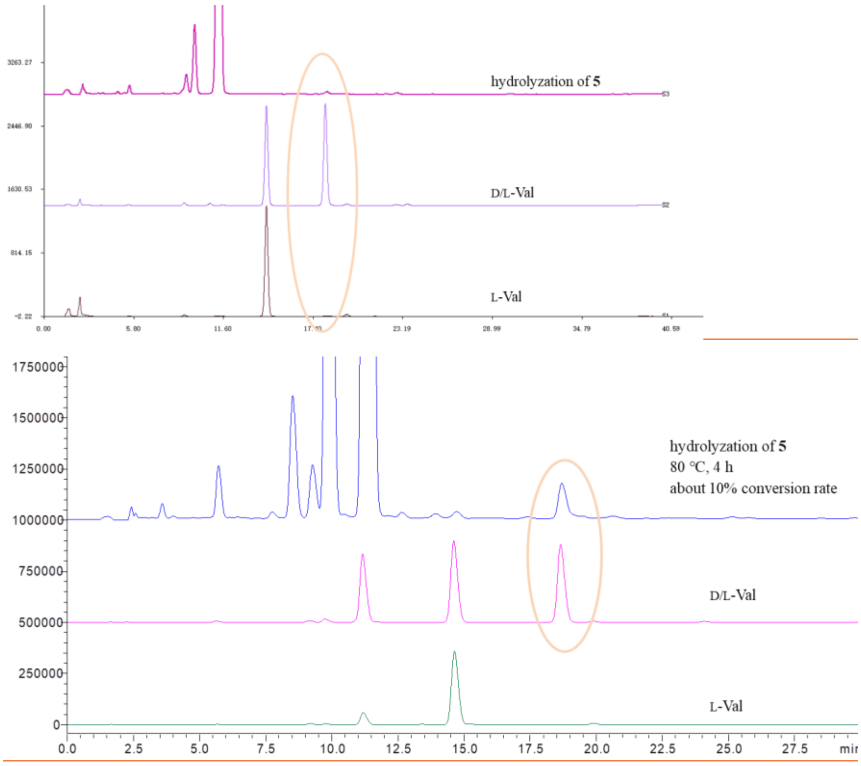

Figure S27. Marfey's analysis of the valine acid in **5** on HPLC

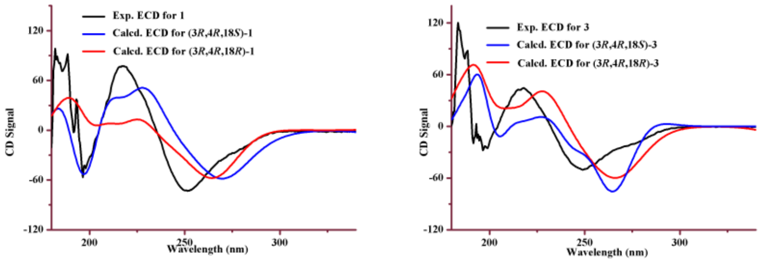

Figure S28. Experimental and calculated ECD spectra of **1** and **3**

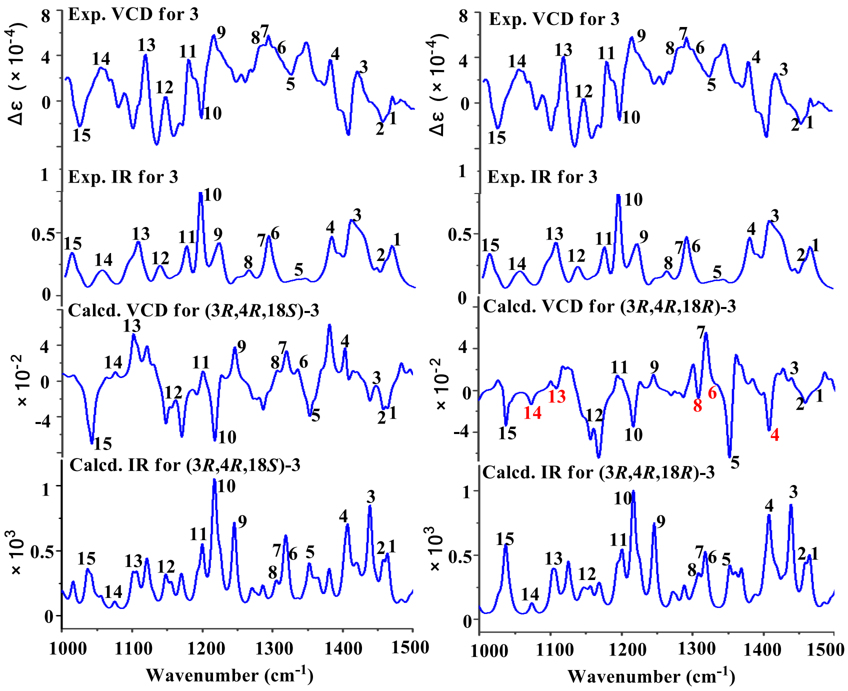

Figure S29. Experimental and calculated VCD/IR spectra of **3**

Table S1. The coordinate for the lowest-energy conformer [(3*R*,15*R*,19*S*)**-1**] in ECD calculations

	Coordinates (Angstroms) X	Y	Z
C	1.45499800	-0.90622200	0.61526600

C	1.99170600	0.22810800	0.05695900
C	1.05784800	1.23961400	-0.42105800
N	-0.32150300	0.90904300	-0.25547600
C	-0.70239900	-0.31940600	0.19324400
N	0.14056300	-1.20883000	0.63550600
O	2.24304000	-1.83483700	1.26958100
C	3.20674200	-2.45493600	0.49063400
C	4.20635100	-1.81929700	-0.12095000
C	4.39882500	-0.37258600	-0.14712400
C	3.40383100	0.54285200	-0.05974600
C	-1.30478800	1.95470300	-0.62690700
C	-2.52723700	1.32803400	-1.28444900
N	-2.83473300	0.05926800	-0.87571000
C	-2.19842000	-0.65873500	0.19709700
O	5.71034800	-0.06785600	-0.34711500
C	6.08287200	1.29928100	-0.47346100
O	-3.19596700	1.92289200	-2.10858300
O	1.38051100	2.31495200	-0.90327900
C	-2.45818300	-2.18378800	0.02850300
C	-1.73685700	2.86478200	0.57483900
C	-2.45301400	4.11696400	0.04974100
C	-0.56180100	3.26680500	1.47525400
C	-1.91126300	-2.73080400	-1.30706600
C	-3.95056700	-2.51284000	0.20705600
C	-1.88482700	-4.26123100	-1.37443100
O	-2.77662900	-0.14625600	1.42222900
C	-2.37549500	-0.73020000	2.66191300
H	3.09368600	-3.53377700	0.47086900
H	4.94366700	-2.41731600	-0.64595500
H	3.61855500	1.59537000	-0.18737500
H	-0.80751700	2.57119500	-1.37340900
H	-3.71700100	-0.29339200	-1.22054400
H	7.15969600	1.29881900	-0.62983500
H	5.58587800	1.76504500	-1.32976100
H	5.84404300	1.85847700	0.43620500
H	-1.89580000	-2.66775500	0.83024900
H	-2.44046300	2.28385000	1.17827600
H	-2.81294600	4.71683000	0.89046000
H	-3.30580200	3.87705000	-0.58535700
H	-1.76922500	4.74187700	-0.53385800
H	-0.93423500	3.88627700	2.29596100
H	-0.06272700	2.40503900	1.92509900
H	0.18730500	3.84326500	0.92925800
H	-2.50931800	-2.33970400	-2.13771700
H	-0.89513900	-2.36110100	-1.46298100
H	-4.38645900	-2.00811000	1.06948400
H	-4.08794800	-3.58646000	0.34687000
H	-4.54322600	-2.23567600	-0.67219500
H	-1.43463900	-4.59671300	-2.31254900
H	-1.29290100	-4.68385200	-0.55656700
H	-2.88508800	-4.69902000	-1.32299700
H	-2.81828100	-0.10293000	3.43529900
H	-2.75495700	-1.75035800	2.77541700
H	-1.28981000	-0.74105400	2.78286600

Table S2. The coordinate for the lowest-energy conformer [(3*R*,15*R*,19*R*)**-1**] in ECD calculations

	Coordinates (Angstroms)		
	X	Y	Z
C	1.40589500	-1.26740200	-0.06729600
C	2.06606400	-0.09993100	-0.36428200
C	1.25490000	1.09355800	-0.55906000
N	-0.14473800	0.91205400	-0.33852800

C	-0.66478000	-0.32363300	-0.10139100
N	0.06722600	-1.39030100	0.05264900
O	2.06825200	-2.47475700	0.03081400
C	3.07575300	-2.54703900	0.97961200
C	4.17102400	-1.78857800	0.98348500
C	4.44654600	-0.69813600	0.05109000
C	3.49921300	0.05415500	-0.55574700
C	-0.98996300	2.12843600	-0.41082000
C	-2.34206700	1.80840100	-1.03471100
N	-2.79875300	0.53429100	-0.83867200
C	-2.18881100	-0.45748500	0.00715800
O	5.78739900	-0.49502900	-0.06518800
C	6.24717700	0.57323600	-0.88452100
O	-2.98195500	2.63823800	-1.65301800
O	1.68900800	2.18700500	-0.88930600
C	-2.69258700	-1.87215400	-0.40318800
C	-1.18643600	2.84206800	0.97265500
C	-1.70887800	4.26838800	0.75012500
C	0.08969800	2.87224800	1.82344200
C	-4.20812300	-2.02239600	-0.13779400
C	-2.33917300	-2.22076400	-1.85713700
C	-4.66635200	-3.48118700	-0.03495500
O	-2.57370200	-0.11777200	1.36218900
C	-2.16226900	-0.97913100	2.42241700
H	2.90415400	-3.33844900	1.70161700
H	4.92498600	-1.98673700	1.73799200
H	3.78124700	0.90845600	-1.15627600
H	-0.46766100	2.80578100	-1.08374200
H	-3.74824300	0.37284900	-1.14576700
H	7.33329200	0.54529500	-0.82478600
H	5.93235500	0.43605800	-1.92336500
H	5.88214600	1.53677000	-0.51622700
H	-2.15754400	-2.57486100	0.23977700
H	-1.94173700	2.27198600	1.52148700
H	-1.92169100	4.73976900	1.71380100
H	-2.62065800	4.29137200	0.15311300
H	-0.96025800	4.88250600	0.23928500
H	-0.12439600	3.37155400	2.77255200
H	0.45879500	1.87210900	2.06398100
H	0.89469200	3.41844200	1.32831000
H	-4.47668900	-1.49896500	0.78291600
H	-4.77773400	-1.53525300	-0.94041100
H	-2.68488100	-3.22872900	-2.09610700
H	-1.26329700	-2.20318100	-2.03045300
H	-2.81147500	-1.53474300	-2.56551300
H	-5.73744400	-3.53617000	0.17713400
H	-4.49055400	-4.03967000	-0.95773200
H	-4.14469300	-4.00528900	0.77204400
H	-2.44707500	-0.46683700	3.34116300
H	-2.67239600	-1.94647800	2.38538600
H	-1.08239600	-1.14730600	2.42324100

Table S3. The coordinate for the lowest-energy conformer [(3*R*,15*R*,19*S*)-**3**] in ECD calculations

	Coordinates (Angstroms)		
	X	Y	Z
C	-1.46585200	-1.19631000	-0.32647600
C	-1.93094500	-0.04119100	0.25793500
C	-0.94697800	0.97278100	0.59800700
N	0.40014100	0.66671600	0.21738800
C	0.71857800	-0.55167700	-0.28100800
N	-0.16887400	-1.46175600	-0.57580300

O	-2.29602600	-2.25910400	-0.61172000
C	-3.37844000	-2.00102000	-1.43928600
C	-4.35263100	-1.13290700	-1.17293200
C	-4.39821100	-0.23813900	-0.01884600
C	-3.30972300	0.24911000	0.61992600
C	1.37503900	1.77439400	0.37910300
C	2.81707400	1.30000700	0.40732100
N	3.08226800	0.04133600	-0.03991800
C	2.17247000	-0.93880000	-0.55954400
O	-5.68375700	0.09686300	0.27669200
C	-5.92269700	1.01713800	1.33491200
O	3.70099200	2.03285500	0.81632100
O	-1.18829600	2.02760100	1.16645100
C	2.49103000	-2.35875000	0.01284500
C	1.14476300	2.88294100	-0.70231800
C	1.68055700	2.50294000	-2.08730200
C	1.70216800	4.23107400	-0.23070000
C	2.18008000	-2.54373800	1.51832700
C	3.91270900	-2.80766600	-0.35361500
C	3.13735100	-1.91953100	2.54168100
O	2.33808400	-0.94663300	-1.98951700
H	-3.37775100	-2.63394700	-2.32018100
H	-5.18363000	-1.07325500	-1.86785000
H	-3.42030100	0.98752200	1.40265800
H	1.16645200	2.21558400	1.35318800
H	4.06453800	-0.19883500	-0.04748200
H	-5.54827000	0.62829600	2.28666300
H	-5.45679500	1.98511000	1.12682300
H	-7.00325800	1.13371700	1.38714300
H	1.79266300	-3.01637800	-0.51350200
H	0.05874700	2.98716500	-0.76657700
H	1.37091500	3.25335900	-2.81986900
H	1.31346000	1.53424200	-2.43346200
H	2.77355000	2.46419200	-2.10273500
H	1.46455700	5.00563300	-0.96557000
H	1.25925400	4.53073600	0.72290100
H	2.78550500	4.20027500	-0.10481800
H	1.16054000	-2.19961100	1.71720100
H	2.15041900	-3.62534500	1.69361300
H	4.04914700	-3.85665200	-0.07736600
H	4.09998900	-2.71606800	-1.42419300
H	4.69013400	-2.24612800	0.17265600
H	2.81044300	-2.17490500	3.55349700
H	4.15851400	-2.29492700	2.43473400
H	3.17747600	-0.83079100	2.48027500
H	1.72293700	-1.59965700	-2.35340300

Table S4. The coordinate for the lowest-energy conformer [(3*R*,15*R*,19*R*)-**3**] in ECD calculations

	Coordinates (Angstroms)		
	X	Y	Z
C	1.43770600	-1.00793400	0.65899300
C	2.00595400	0.09792000	0.07139000
C	1.10155900	1.13404300	-0.40253900
N	-0.29040700	0.86337500	-0.20174000
C	-0.70237500	-0.34007800	0.26442400
N	0.11486200	-1.25700100	0.70423900
O	2.19937500	-1.95476000	1.31429900
C	3.13556500	-2.61854300	0.53681200

C	4.14788000	-2.02723300	-0.09733600
C	4.38877500	-0.58837400	-0.15279100
C	3.42667400	0.36150800	-0.07105900
C	-1.20922600	1.98907600	-0.50130500
C	-2.64115900	1.54217000	-0.73883800
N	-2.99179100	0.28946000	-0.33221300
C	-2.18719500	-0.69956900	0.32835200
O	5.70772600	-0.33270500	-0.37160400
C	6.12679000	1.01868200	-0.51940800
O	-3.44323600	2.29105100	-1.26842400
O	1.44516900	2.19342300	-0.90609900
C	-2.44283300	-2.10592100	-0.30245500
C	-1.11842100	3.09477300	0.60388900
C	-1.86444200	2.73015400	1.89223100
C	-1.56743700	4.45385500	0.05454400
C	-3.92460100	-2.51681200	-0.16892800
C	-1.95689500	-2.18012200	-1.75565800
C	-4.14992600	-4.02855000	-0.28015000
O	-2.56553400	-0.70270600	1.71436800
H	2.98774000	-3.69322000	0.53947200
H	4.85907500	-2.65922200	-0.61858500
H	3.67573200	1.40385900	-0.21877300
H	-0.85199200	2.42493900	-1.43375200
H	-3.97119300	0.07001900	-0.45875800
H	7.20144200	0.97831400	-0.68504300
H	5.63887000	1.49063500	-1.37757500
H	5.91589600	1.59739200	0.38491800
H	-1.84109700	-2.79815100	0.29513600
H	-0.05218800	3.17360500	0.83072300
H	-1.64793600	3.47247100	2.66532700
H	-1.57803500	1.75254300	2.28600600
H	-2.94814400	2.72064700	1.74441400
H	-1.43128900	5.22421100	0.81907700
H	-0.97580600	4.74351700	-0.81809000
H	-2.61854700	4.44514000	-0.23814300
H	-4.30820500	-2.17507300	0.79622700
H	-4.52047500	-2.01599400	-0.94309700
H	-2.14130000	-3.17257900	-2.17194300
H	-0.88382600	-1.99466100	-1.84216200
H	-2.47884500	-1.45605300	-2.38759800
H	-5.20964800	-4.27094900	-0.16432600
H	-3.83075400	-4.42814000	-1.24627100
H	-3.60277000	-4.56846300	0.49880000
H	-2.00171100	-1.34433000	2.17067200

Table S5. The coordinate for the lowest-energy conformer [(3*R*,15*R*,19*S*)-**1**] in VCD calculations

	Coordinates (Angstroms) X	Y	Z
C	1.45589900	-0.92089000	0.61573800
C	1.99601600	0.22471300	0.07220300
C	1.06093800	1.24584100	-0.38980100
N	-0.31878300	0.90881300	-0.24839800
C	-0.70409700	-0.32619400	0.18665700
N	0.13723400	-1.22446500	0.62715300
O	2.24673500	-1.85888900	1.26321600
C	3.19618400	-2.47622700	0.45645800
C	4.20102600	-1.83469700	-0.15405100
C	4.40522100	-0.38507400	-0.15249100
C	3.41182900	0.53826300	-0.04068500
C	-1.29727800	1.95469000	-0.63525300

C	-2.52265900	1.33226900	-1.29273800
N	-2.82299100	0.04991000	-0.90847700
C	-2.20305000	-0.65797500	0.18484400
O	5.72241700	-0.08546000	-0.35933300
C	6.09476400	1.28884900	-0.47041400
O	-3.20604800	1.94500100	-2.10516200
O	1.38935000	2.34172600	-0.84581500
C	-2.47547800	-2.18401300	0.03898500
C	-1.74359400	2.88756100	0.54795900
C	-2.32622000	4.19342100	-0.01965400
C	-0.61344000	3.19466800	1.54304400
C	-1.93539900	-2.75540700	-1.29193600
C	-3.97332700	-2.49842500	0.22617700
C	-1.92218700	-4.29063000	-1.33853800
O	-2.78647300	-0.11649200	1.39749200
C	-2.37908100	-0.67280300	2.65401000
H	3.06979500	-3.55543300	0.40687900
H	4.92811100	-2.43031500	-0.69954300
H	3.62970700	1.59369500	-0.15339200
H	-0.79269300	2.56311200	-1.38578900
H	-3.72055500	-0.28698700	-1.24069300
H	7.17264000	1.29054900	-0.63792500
H	5.58868400	1.76784600	-1.31690200
H	5.86532700	1.83611200	0.45148900
H	-1.91535300	-2.66299500	0.84655900
H	-2.53198100	2.35918500	1.09421800
H	-2.72645900	4.80538000	0.79620000
H	-3.12952700	4.00885600	-0.73636900
H	-1.54746100	4.77694400	-0.52489100
H	-1.00542100	3.83677800	2.33946300
H	-0.22116800	2.29109200	2.02047300
H	0.21821100	3.71671600	1.06214700
H	-2.53261800	-2.37078600	-2.12788300
H	-0.91407400	-2.39851700	-1.45262200
H	-4.39930600	-1.98223300	1.08954900
H	-4.11710400	-3.57200700	0.37402100
H	-4.56626100	-2.22060700	-0.65462000
H	-1.47586500	-4.63992900	-2.27568500
H	-1.33023100	-4.70531600	-0.51439000
H	-2.92824700	-4.71809500	-1.27875500
H	-2.82463400	-0.02750300	3.41431800
H	-2.75490500	-1.69367400	2.79154700
H	-1.29109300	-0.67639600	2.77436900

Table S6. The coordinate for the lowest-energy conformer [(3*R*,15*R*,19*R*)**-1**] in VCD calculations

	Coordinates (Angstroms)		
	X	Y	Z
C	1.40531400	-1.28038800	-0.09731200
C	2.06871800	-0.10520800	-0.37718100
C	1.25518900	1.09128600	-0.56405900
N	-0.14442400	0.91033400	-0.34662000
C	-0.66821800	-0.32802100	-0.11361200
N	0.06288600	-1.40233600	0.02844700
O	2.07041800	-2.49362700	-0.02256800
C	3.06286100	-2.57424200	0.94790400
C	4.16320900	-1.81252800	0.97528100
C	4.45121000	-0.70958000	0.05481400
C	3.50589400	0.05106200	-0.55831400
C	-0.98792800	2.12992100	-0.41695800
C	-2.34635600	1.81374100	-1.03184800
N	-2.79964100	0.53141500	-0.85627900
C	-2.19372800	-0.45660400	0.00203200

O	5.79859700	-0.50278600	-0.04026800
C	6.26022400	0.58700400	-0.83967800
O	-2.99884200	2.65753100	-1.63573800
O	1.69562100	2.19510000	-0.88646900
C	-2.70649800	-1.87386700	-0.39286100
C	-1.17658500	2.85171000	0.96518800
C	-1.66593900	4.29226500	0.73514600
C	0.09503900	2.85544200	1.82932100
C	-4.22197400	-2.01732000	-0.11010800
C	-2.36881300	-2.23561900	-1.85070000
C	-4.68356800	-3.47742300	0.01411000
O	-2.57394200	-0.09849300	1.35591500
C	-2.14426000	-0.94731000	2.42630500
H	2.87890500	-3.36827500	1.66818800
H	4.90599900	-2.01789600	1.74155600
H	3.79268200	0.91638000	-1.14389000
H	-0.46905300	2.80633300	-1.09621200
H	-3.76041400	0.38141700	-1.14611000
H	7.34853100	0.56594900	-0.76864200
H	5.95758000	0.46467200	-1.88630600
H	5.88369700	1.54356700	-0.45832200
H	-2.16685400	-2.57571000	0.24878800
H	-1.94900400	2.30068000	1.51180900
H	-1.88157700	4.76651400	1.69884900
H	-2.57041000	4.33098700	0.12432700
H	-0.89419000	4.88866600	0.23426800
H	-0.12008700	3.36380800	2.77559100
H	0.43658600	1.84509600	2.07619200
H	0.91729300	3.38242400	1.33780700
H	-4.47937300	-1.48433900	0.81023800
H	-4.79877400	-1.53893600	-0.91446200
H	-2.72190800	-3.24650900	-2.07468000
H	-1.29236700	-2.22231300	-2.03294900
H	-2.84770600	-1.55168700	-2.55940600
H	-5.75533900	-3.52464400	0.23421600
H	-4.51341600	-4.04637600	-0.90536400
H	-4.15523800	-3.99124700	0.82569400
H	-2.41880500	-0.42050600	3.34294900
H	-2.65408700	-1.91791300	2.40974700
H	-1.06198700	-1.11376300	2.41548400

Table S7. The coordinate for the lowest-energy conformer [(3*R*,15*R*,19*S*)-**3**] in VCD calculations

	Coordinates (Angstroms)		
	X	Y	Z
C	1.45566400	-0.81495600	0.82209300
C	1.90864700	0.27901500	0.11389700
C	0.90003600	1.17892000	-0.43113700
N	-0.45712000	0.79771200	-0.18038000
C	-0.74869800	-0.39210900	0.40602800
N	0.15930300	-1.18355500	0.91882100
O	2.31811100	-1.61792600	1.55062700
C	3.28196100	-2.27804000	0.79542300
C	4.22804300	-1.66407600	0.07352200
C	4.34381500	-0.21697200	-0.11682700
C	3.29936000	0.65455000	-0.09077700
C	-1.48403300	1.79792200	-0.56932600
C	-2.87008700	1.19607900	-0.73397300
N	-3.09407900	-0.04727300	-0.21484300
C	-2.19364600	-0.88485700	0.53172300
O	5.63526000	0.12768400	-0.40087900

C	5.92337700	1.49692700	-0.68735700
O	-3.75384500	1.81896100	-1.31507400
O	1.13822200	2.22369000	-1.03820400
C	-2.31499000	-2.36723400	0.04826400
C	-1.48978300	3.01265600	0.41953200
C	-2.20110300	2.71631800	1.74861500
C	-2.05836100	4.26817900	-0.26027100
C	-1.87089600	-2.53516300	-1.41959300
C	-3.73186400	-2.91686300	0.29594600
C	-1.76312800	-3.99868800	-1.87312100
O	-2.55924500	-0.78293500	1.92013200
H	3.22085200	-3.35991500	0.88877700
H	4.97493600	-2.28299200	-0.41648600
H	3.45006500	1.69743400	-0.34370900
H	-1.18278300	2.17252000	-1.54877000
H	-4.05420800	-0.36589000	-0.30036200
H	6.99588900	1.53894900	-0.88168000
H	5.37416100	1.83931700	-1.57232700
H	5.67916900	2.13874600	0.16729600
H	-1.61527600	-2.92864400	0.67726800
H	-0.43401600	3.20864300	0.62761800
H	-2.04294900	3.54983200	2.44138200
H	-1.82986500	1.80763000	2.23211900
H	-3.28190100	2.60249400	1.61146600
H	-1.98555400	5.12100200	0.42368900
H	-1.49339500	4.51548700	-1.16537700
H	-3.10726300	4.13597600	-0.53761000
H	-2.56931300	-1.99999000	-2.07497400
H	-0.89188300	-2.06262600	-1.56124200
H	-3.73731300	-4.00599200	0.20043300
H	-4.08721000	-2.66947300	1.29960800
H	-4.45670200	-2.53364200	-0.43351800
H	-1.37102700	-4.05451800	-2.89401600
H	-1.08425200	-4.56565100	-1.22537400
H	-2.73309400	-4.50540200	-1.86868100
H	-1.90770500	-1.30693100	2.42050900

Table S8. The coordinate for the lowest-energy conformer [(3*R*,15*R*,19*R*)-**3**] in VCD calculations

	Coordinates (Angstroms)		
	X	Y	Z
C	1.44087400	-1.00956300	0.66610500
C	2.01160900	0.10287100	0.08264400
C	1.10510800	1.14399400	-0.38435600
N	-0.28737300	0.86889200	-0.19788800
C	-0.70301800	-0.34156600	0.25702400
N	0.11400800	-1.26308800	0.70087100
O	2.20557300	-1.95776500	1.32615400
C	3.12977100	-2.62550400	0.52923300
C	4.14757800	-2.03306100	-0.10823400
C	4.39774200	-0.59104700	-0.15082600
C	3.43559500	0.36622100	-0.05715400
C	-1.20586600	1.99615900	-0.49946500
C	-2.63501800	1.54718400	-0.75526400
N	-2.98427000	0.28039900	-0.38063400
C	-2.19072900	-0.70033900	0.31139800
O	5.72210700	-0.34043000	-0.37598000
C	6.13897700	1.01773200	-0.52129800
O	-3.44174200	2.30647600	-1.28357700
O	1.45442300	2.21815300	-0.87539600
C	-2.44386700	-2.11789300	-0.29735300

C	-1.13106000	3.09844200	0.61143400
C	-1.90960900	2.73678600	1.88550200
C	-1.55935600	4.46505200	0.05436400
C	-3.92788700	-2.52857800	-0.15874300
C	-1.95328200	-2.21589300	-1.75090800
C	-4.15336500	-4.04543800	-0.25265800
O	-2.58350800	-0.67722700	1.69684700
H	2.97027100	-3.70121500	0.51280800
H	4.85055700	-2.66696100	-0.64207300
H	3.68651700	1.41056900	-0.20092100
H	-0.84171000	2.43984000	-1.42733300
H	-3.96726400	0.06485600	-0.51506000
H	7.21521900	0.97978500	-0.69483400
H	5.64381700	1.49377800	-1.37592700
H	5.93360000	1.59327700	0.38899800
H	-1.84222100	-2.80142800	0.31192900
H	-0.06895500	3.17061700	0.86277500
H	-1.70334400	3.48027500	2.66291600
H	-1.63534300	1.75515000	2.28332700
H	-2.99083800	2.73430500	1.71051200
H	-1.43696600	5.23109900	0.82805600
H	-0.94076200	4.75217500	-0.80260000
H	-2.60465600	4.46019700	-0.26491900
H	-4.31077400	-2.17698100	0.80498500
H	-4.52464400	-2.03792500	-0.94040300
H	-2.13310500	-3.21953200	-2.14686300
H	-0.87893900	-2.02638500	-1.83520600
H	-2.47852700	-1.50391400	-2.39675000
H	-5.21538600	-4.28402900	-0.13226300
H	-3.83418400	-4.45422300	-1.21685300
H	-3.60319000	-4.57514500	0.53354700
H	-2.00572900	-1.30861800	2.16264100
